# Insulin and IGF1 Receptors Are Essential for XX and XY Gonadal Differentiation and Adrenal Development in Mice

**DOI:** 10.1371/journal.pgen.1003160

**Published:** 2013-01-03

**Authors:** Jean-Luc Pitetti, Pierre Calvel, Yannick Romero, Béatrice Conne, Vy Truong, Marilena D. Papaioannou, Olivier Schaad, Mylène Docquier, Pedro Luis Herrera, Dagmar Wilhelm, Serge Nef

**Affiliations:** 1 Department of Genetic Medicine and Development, University of Geneva Medical School, University of Geneva, Geneva, Switzerland; 2 Division of Molecular Genetics and Development, Institute for Molecular Bioscience, The University of Queensland, Brisbane, Australia; 3 Genomics Platform, National Center of Competence in Research “Frontiers in Genetics,” University of Geneva, Geneva, Switzerland; National Institute of Environmental Health Sciences, United States of America

## Abstract

Mouse sex determination provides an attractive model to study how regulatory genetic networks and signaling pathways control cell specification and cell fate decisions. This study characterizes in detail the essential role played by the insulin receptor (INSR) and the IGF type I receptor (IGF1R) in adrenogenital development and primary sex determination. Constitutive ablation of insulin/IGF signaling pathway led to reduced proliferation rate of somatic progenitor cells in both XX and XY gonads prior to sex determination together with the downregulation of hundreds of genes associated with the adrenal, testicular, and ovarian genetic programs. These findings indicate that prior to sex determination somatic progenitors in *Insr;Igf1r* mutant gonads are not lineage primed and thus incapable of upregulating/repressing the male and female genetic programs required for cell fate restriction. In consequence, embryos lacking functional insulin/IGF signaling exhibit (i) complete agenesis of the adrenal cortex, (ii) embryonic XY gonadal sex reversal, with a delay of *Sry* upregulation and the subsequent failure of the testicular genetic program, and (iii) a delay in ovarian differentiation so that *Insr;Igf1r* mutant gonads, irrespective of genetic sex, remained in an extended undifferentiated state, before the ovarian differentiation program ultimately is initiated at around E16.5.

## Author Summary

Congenital disorders of sexual differentiation are rare diseases in which there is discordance between chromosomal, gonadal, and phenotypic sex. Unfortunately, only a minority of patients clinically diagnosed with disorders of sex development (DSD) obtains a molecular diagnosis, indicating that our understanding of the factors and signaling pathways mediating gonadal development and sex determination is far from complete. Using mouse models, we show that the insulin receptor (INSR) and the IGF type I receptor (IGF1R) are required to mediate different aspects of adrenogonadal development such as adrenal specification, testicular differentiation, and ovarian development. We found that a complex dynamic transcriptional program is initiated in somatic progenitor cells of the bipotential gonadal primordium prior to sex determination. A significant fraction of this genetic program is prematurely altered in the somatic progenitors lacking insulin/IGF signaling, which explains adrenal agenesis and the incapacity of XX and XY mutant gonads to develop into ovaries or testes. This finding sheds light on a crucial, but so far underestimated, signaling pathway underlying sex determination in mice and potentially DSDs in humans.

## Introduction

Both the gonads and the adrenal cortex originate from a common structure referred to as the adreno-genital primordium (AGP). In mice, the AGP is visible at embryonic day (E) 9 [1], and is composed of a population of precursor cells expressing the nuclear receptor steroidogenic factor 1 (SF1, also named Ftzf1 or Ad4BP; [2]). As development proceeds, the AGP separates into two distinct regions [2]. The adrenocortical primordium separates from the gonadal primordium in the rostral region of the AGP at around E10.5, and differentiates into the adrenal cortex in both sexes, ultimately giving rise to the zona glomerula, fascicula and reticularis. In parallel, the bipotential gonadal primordium, composed of primordial germ cells and SF1-positive somatic cells, differentiates into a testis or an ovary depending on the genetic sex. Gonadal differentiation is controlled by a balance of antagonistic pathways. In XY individuals, testis development is initiated by the transient expression of SRY, which, in concert with SF1, triggers *Sox9* upregulation, leading to Sertoli cell commitment and testicular differentiation [3]. Sertoli cell differentiation is a result of the establishment of a positive feedback loop between SOX9 and FGF9 as well as SOX9 and PGD_2_ secretion [4], [5]. SOX9/FGF9 also act antagonistically by down regulating female signals such as WNT4 thereby blocking ovarian differentiation [6]. In XX individuals, the bipotential gonad develops as an ovary. Although no morphological differentiation is apparent up until E13.5 when germ cells enter meiosis under the influence of retinoic acid [7], [8], a robust ovarian-specific genetic program is initiated as early as E11.5 [9], [10]. The R-spondin1/Wnt4/β-catenin pathway and the transcription factor FOXL2 have been shown to act in a complementary manner to promote ovarian development and antagonize the testicular pathway by silencing *Sox9* and *Fgf9* (reviewed in [11]).

As the AGP is the common precursor of both the adrenal cortex and the gonads, mutations in genes important for its initial specification and differentiation usually manifest themselves as defects in the development of both adrenal and gonadal tissues [12]. For example, targeted inactivation of the orphan nuclear receptor SF1 [13], the Wilms' tumor-suppressor WT1 [14], the polycomb factor M33 (CBX2; [15]), the transcription co-factor Cited2 [16] , the homeodomain protein PBX1 [17], and the transcription factor Odd-skipped related 1 (ODD1; [18]) lead to adrenal agenesis, impaired thickening of the genital ridges, and subsequent gonadal degeneration and XY sex reversal. Nevertheless, our understanding of the molecular pathways that direct adrenal cortex and gonad development and differentiation remains incomplete, and it has become clear that additional factors and signaling pathways must be involved.

Insulin and its related growth factors IGF1 and IGF2 modulate a variety of physiological activities including metabolism, stimulation of cell proliferation, differentiation and survival [19]. The action of these growth factors on target cells is mediated by the insulin receptor (INSR) and the IGF type I receptor (IGF1R), two membrane-associated tyrosine kinase receptors. Insulin and IGF1 bind primarily to INSR and IGF1R respectively, while IGF2 seems to act through either IGF1R or the A isoform of INSR (for review see [20]).

In recent years, increasing evidence has emerged that the insulin family of growth factors plays an essential role in gonadal development and sex determination. Of particular importance is the observation that insulin/IGF signaling is absolutely required for testis differentiation in mice [21]. However it remains unclear whether the insulin/IGF signaling pathway acts upstream of SRY by affecting adrenogenital precursor cells, or whether it influences *Sry* expression and the male transcriptional program directly in Sertoli cell precursors. Furthermore, there have been no studies addressing a potential role for insulin/IGF signaling in ovarian differentiation and adreno-cortical development.

## Results

### Homozygous deletion of *Insr* and *Igf1r* causes male-to-female sex reversal

**Figure 1:**
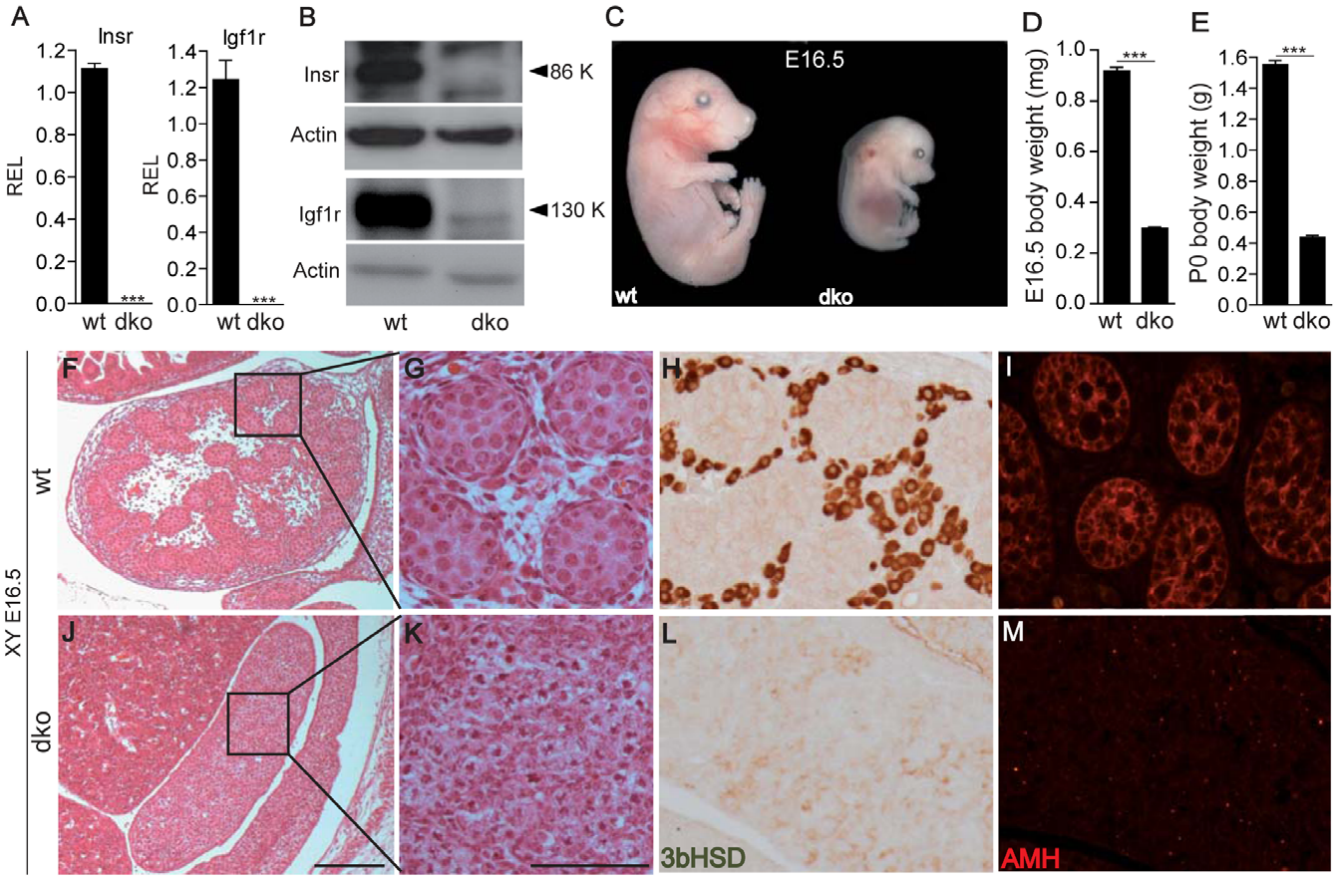
Growth retardation and sex reversal in *Insr* ^Δ/Δ^;*Igf1r* ^Δ/Δ^ (dko) embryos. Quantitative RT-PCR (A) and a representative Western blot (B) show a complete absence of *Insr* and *Igf1r* transcripts and proteins in mutant whole embryos (*Insr* ^Δ/Δ^;*Igf1r* ^Δ/Δ^, or dko) compared to controls (wt). Photomicrograph of E16.5 embryos (C) and their respective body weight at E16.5 (D) and at P0 (E) reveal a significant reduction in the body weight of dko animals. F–M Micrographs showing haematoxylin and eosin staining (F,G,J,K), as well as IHC staining for the Leydig cell marker 3βHSD (H,L), and the Sertoli cell marker AMH (I,M), of XY gonads from control (F–I) and dko (J–M) embryos at E16.5. Note the complete absence of seminiferous tubules (K) and male-specific markers (L,M) in dko mutant gonads. REL, relative expression levels. Values are expressed as means ± SEM, ***p<0.001 vs control. Scale bars: 100 µm.

A significant constraint in our past research has been the low recovery frequency of XY *Insr;Igf1r;Irr* triple constitutive ko animals (1/32) due to the lethal phenotype of single constitutive *Insr* and *Igf1r* mutants [21]. To bypass this lethality, and thus generate a large number of constitutive *Insr;Igf1r* double knockout animals, we crossed mice bearing pairs of loxP-flanked alleles of both *Insr* [22] and *Igf1r* [23]; *Insr^fx/fx^;Igf1r^fx/fx^*) with mice carrying either an oocyte-specific *Gdf9:Cre* transgene [24] or a spermatogenesis-specific *Ngn3:Cre* transgene [25]. We found that mice lacking both *Insr* and *Igf1r* in either the male germ line (*Ngn3:Cre;Insr^fx/fx^;Igf1r^fx/fx^)* or in oocytes (*Gdf9:Cre;Insr^fx/fx^;Igf1r^fx/fx^*) have normal reproductive functions (data not shown and [26]). When crossed, these animals produce large numbers of constitutive double knockout embryos (*Insr* ^Δ/Δ;^ *Igf1r* ^Δ/Δ^ or dko), which lack *Insr* and *Igf1r* transcripts and their encoded receptors (Figure 1A, 1B). As previously reported [27], these animals exhibit embryonic growth retardation (68% and 75% of control weight at E16.5 and P0, respectively), edema and dorsal tail flexion (Figure 1C–1E and data not shown). At E16.5, XY dko gonads morphologically resemble ovaries and are histologically indistinguishable from XX gonads, with no evidence of testis cords and complete absence of Sertoli (AMH) and Leydig (3βHSD) cell-specific markers (Figure 1F–1M). In fact, *Insr*;*Igf1r* dko embryos recapitulate the sex-reversed phenotype observed in *Insr;Igf1r;Irr* triple ko animals [21]. This suggested that only *Insr* and *Igf1r*, but not *Irr*, play important roles in testicular differentiation on a mixed genetic background.

### Complete absence of testicular differentiation in XY double mutant gonads

**Figure 2:**
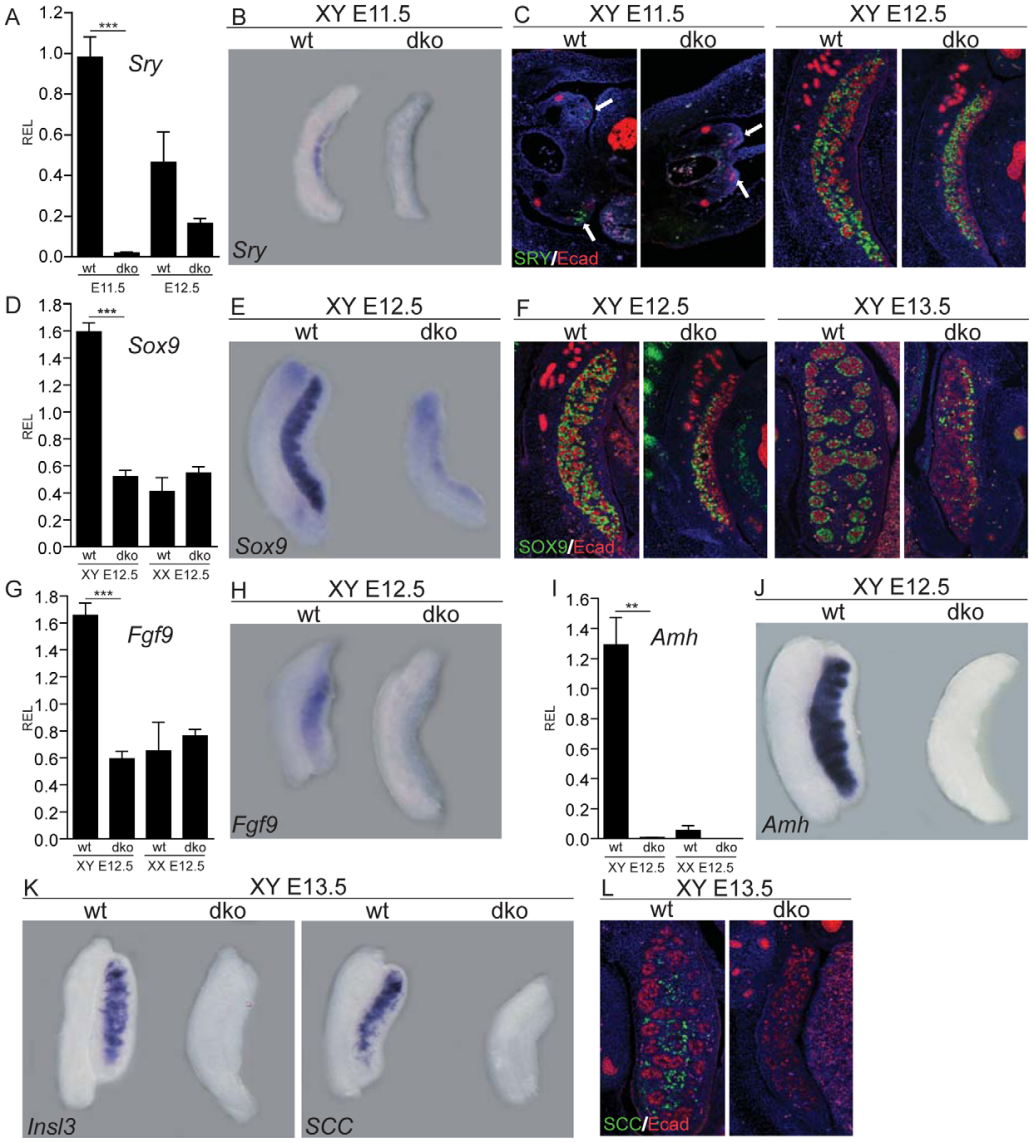
Testicular development is disrupted in the absence of insulin/IGF signaling. Expression of key testicular genes such as *Sry* (A–C), *Sox9* (D–F), *Fgf9* (G,H), *Amh* (I,J), *Insl3* and *p450scc* (K,L) was assessed in XY control and XY dko gonads at E11.5, E12.5 or E13.5 using qRT-PCR (A,D,G,I), whole mount *in situ* hybridization (B,E,H,J,K) or by double immunofluorescence (C,F,L) using sagittal sections, except for the first two panels in C, for which the embryos were cut transversally and arrows point toward genital ridges. XY control and XY dko gonads were immunostained for either SRY (C, in green), the Sertoli-cell marker SOX9 (F, in green), or the Leydig cell marker P450SCC (L, in green), along with E–cadherin (red), which labels germ cells and mesonephric tubules. Note the drastic reduction in *Sry*, *Sox9*, *Fgf9*, *Amh*, *Insl3* and *p450scc* expression and the absence of testis cords in XY dko gonads. REL, relative expression levels. Values are expressed as means ± SEM, **p<0.01, ***p<0.001 vs control.

We next aimed to further dissect the effect of the lack of insulin/IGF signaling on the initiation of the testicular differentiation program. Expression analysis revealed that *Sry* mRNA expression at ∼E11.5 was almost undetectable in dko gonads, which is consistent with an absence of SRY-positive cells in E11.5 dko gonads (Figure 2A–2C). By E12.5, *Sry* transcript and protein were detected in XY dko gonads, but RNA levels were severely reduced compared to controls (Figure 2A, 2C). The expression of *Sox9*, a direct target of SRY whose expression is necessary and sufficient to initiate testis differentiation [28], [29], was significantly reduced in XY dko gonads at E12.5, both at the transcript and protein levels (Figure 2D–2F). By E13.5, only a few SOX9-positive cells were present in XY dko gonads, which coincided with a complete absence of testis cord formation (Figure 2F). The lack of upregulation of SOX9 downstream genes such as *Fgf9* (Figure 2G, 2H), *Amh* (Figure 2I, 2J), and *Ptgds* (data not shown) suggested that SOX9 expression did not reach the threshold necessary for Sertoli cell commitment. As anticipated, differentiation of Leydig cells and steroidogenesis were not initiated, indicated by the absence of Leydig cell-specific markers such as *Insl3* and p450SCC in double mutant gonads at E13.5 and E16.5 (Figure 2K, 2L and data not shown). Overall, this analysis indicates that Sertoli cell differentiation and therefore the initiation of the testis determination program are disrupted in the absence of insulin/IGF signaling.

### Persistence of an uncommitted state and delay in ovarian differentiation in XY and XX gonads lacking insulin/IGF signaling

**Figure 3:**
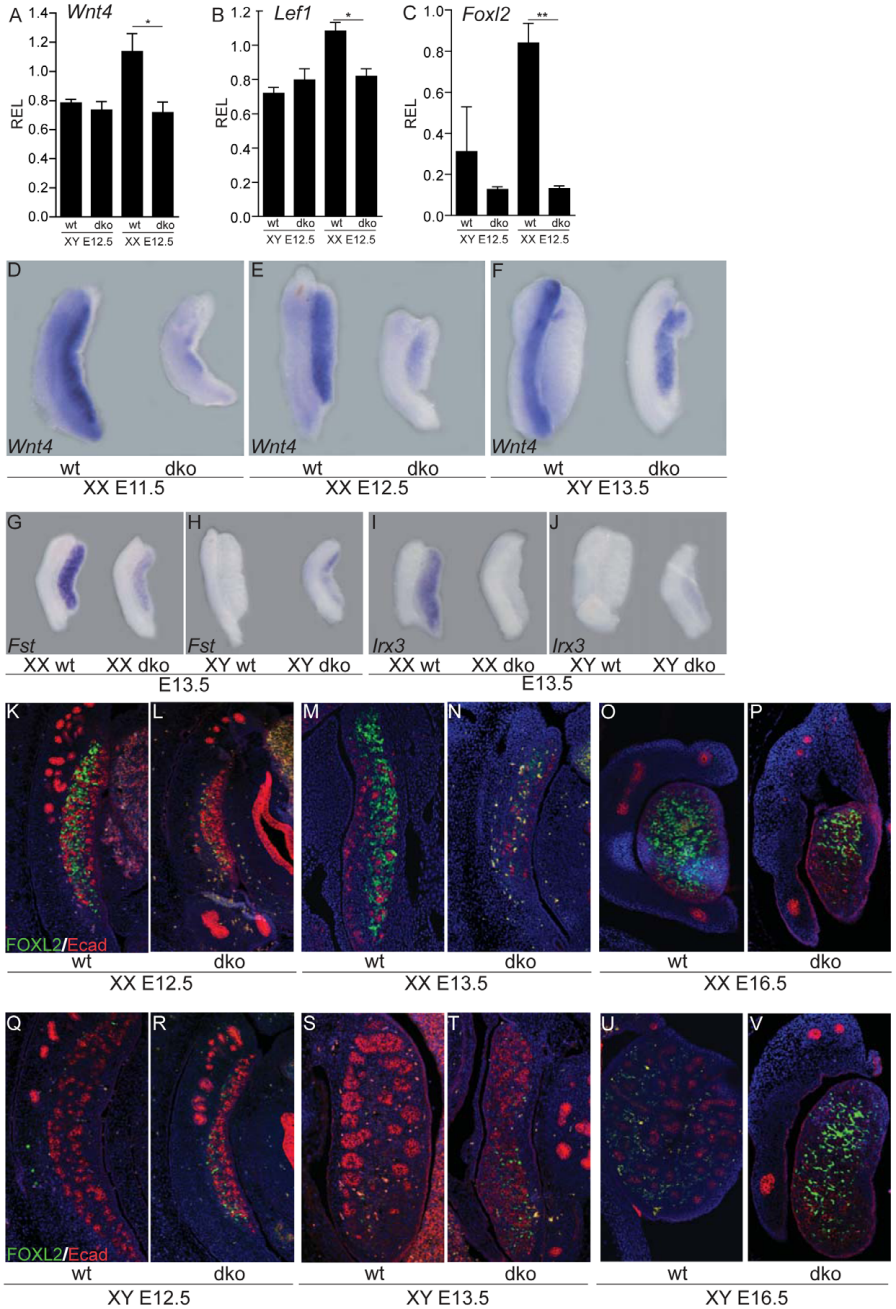
Delay in ovarian differentiation in the absence of insulin/IGF signaling, irrespective of the genetic sex. Expression of key ovarian genes (*Wnt4*, *Fst*, *Irx3*, *Lef1* and *Foxl2*) was assessed in XX or XY genital ridges/mesonephroi either by qRT-PCR (A–C), whole mount *in situ* hybridization (D–J), or with double immunofluorescence using the female marker FOXL2 (green) along with E–cadherin (red; K–V). We observed a significant reduction in levels of *Foxl2*, *Wnt4*, *Fst*, *Irx3* and *Lef1* in E12.5 and E13.5 dko gonads suggesting a delay in ovarian differentiation both in XX and XY dko embryos. REL, relative expression levels. Values are expressed as means ± SEM, *p<0.05, **p<0.01 versus control.

The testicular and ovarian genetic programs are mutually antagonistic such that genital ridges differentiate into either ovaries or testes. Previous loss-of-function studies have shown that in the absence of testicular differentiation, the female program is initiated and ovarian differentiation occurs [29], [30]. Key ovarian-determining components include the R-spondin1/WNT4/β-catenin pathway and the FOXL2 transcription factor, which act in a complementary manner to promote the ovarian fate and repress testicular signaling and development [31], [32], [33], [34]. Developing XX dko gonads (E12.5–E16.5), although reduced in size, were histologically indistinguishable from XX control gonads (data not shown). As expected, none of the testis-specific markers including SOX9 and p450SCC were expressed, indicating that the testicular pathway was never initiated in XX gonads lacking insulin signaling (see Figure S1). However, we found that ovarian differentiation was impaired and delayed in both XY and XX dko gonads (Figure 3). The expression of key ovarian-promoting factors such as *Wnt4* and the downstream genes *Fst* [35] and *Irx3* [36] as well as the nuclear mediator of canonical WNT signaling, *Lef1* [37], were either absent or significantly reduced in XX and XY dko gonads (Figure 3A, 3B, 3D–3J). Similarly, *Foxl2* transcripts and FOXL2-positive cells were drastically reduced or absent at E12.5 and E13.5 in XY and XX double mutant gonads (Figure 3C and 3K–3N, 3Q–3T). By E16.5, we observed FOXL2-positive somatic cells in both XX and XY dko gonads (Figure 3O, 3P, 3U, 3V), indicating that ovarian differentiation had initiated both in XX and XY mutant gonads.

**Figure 4:**
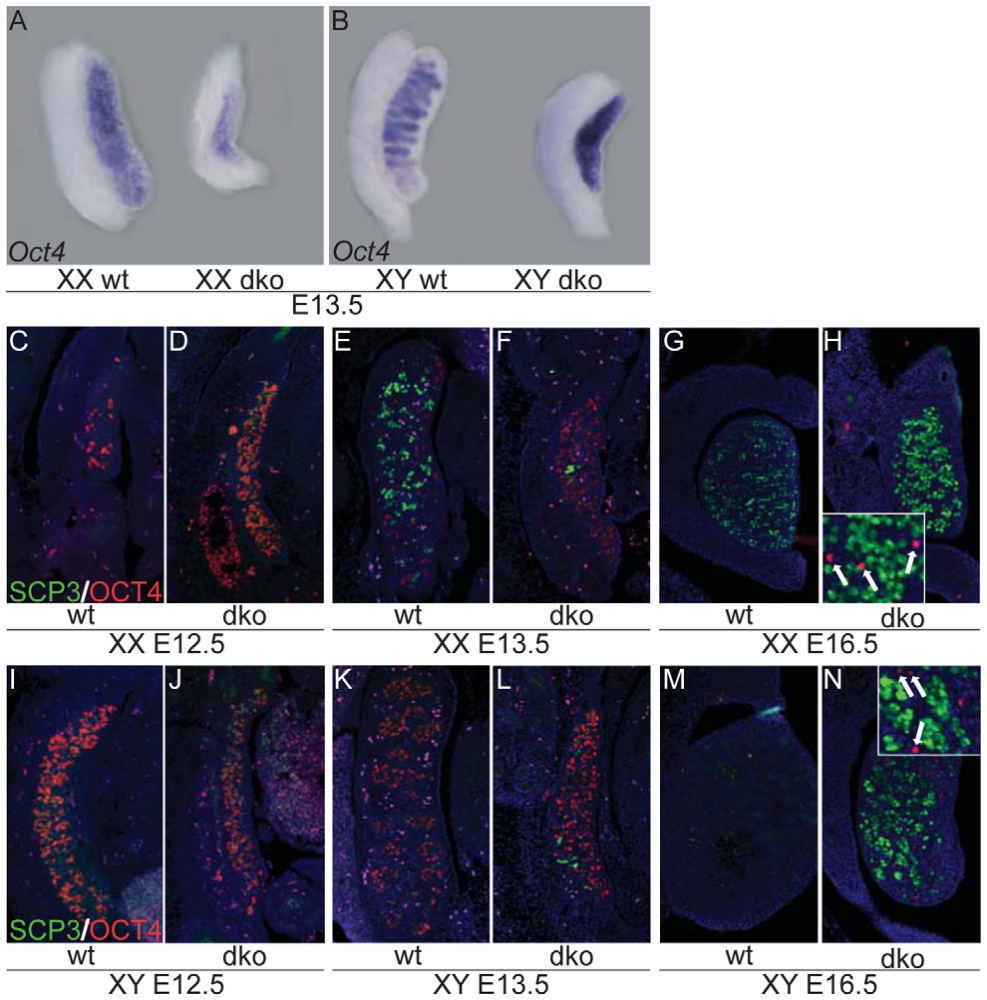
Delayed entry into meiosis in XX and XY gonads lacking *Insr* and *Igf1r*. Expression of germ cell markers was assessed in XX or XY control or dko gonads either by whole mount *in situ* hybridization at E13.5 (A,B), or with double immunofluorescence at E12.5, E13.5 and E16.5 using the meiotic marker SCP3 (green) along with pluripotency marker OCT4 (red; C–N). Note the absence of SCP3 positive cells reflecting delayed progression in meiosis in XX and XY dko gonads at E13.5 (F,L). Arrows in inset H and N point toward few remaining OCT4^+^ germ cells.

We next investigated the germ cell fate in XX and XY dko gonads by comparing the expression of the pluripotency marker OCT4 and the meiotic marker SCP3 (synaptonemal complex protein 3; Figure 4). Approximately the same number of germ cells was present in dko gonads as compared to wild type (Figure 4A, 4B). As expected in XX control gonads, OCT4 was downregulated and SCP3 upregulated in germ cells at E13.5, as they enter meiosis (compare Figure 4C with 4E). In contrast, very few germ cells expressed SCP3 in XX and XY dko gonads at E13.5 (Figure 4F, 4L), indicating a delay in the entry to meiosis. It is only later, at E16.5, that the majority of germ cells in XX and XY dko gonads were SCP3-positive, although a few cells still expressed the pluripotency marker OCT4 (Figure 4H, 4N). Overall, these findings suggest that gonads lacking insulin/IGF signaling, irrespective of the genetic sex, remain in an undifferentiated state for several additional days without clear activation of the testicular or ovarian genetic program.

### Early reduction of SF1^+^ cells in the developing genital ridges of mice lacking *Insr* and *Igf1r*

**Figure 5:**
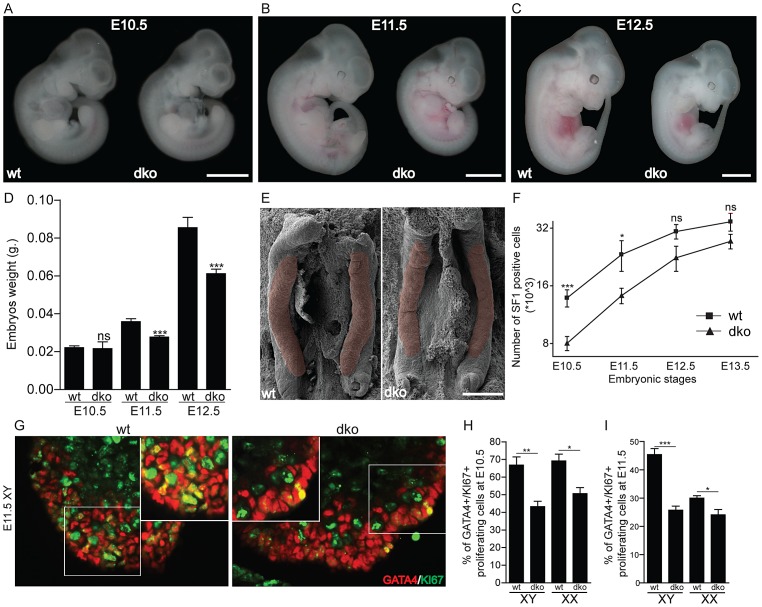
Absence of *Insr* and *Igf1r* significantly reduces proliferation of somatic cell progenitors prior to sex determination. Photomicrographs of control and dko embryos at E10.5 (A, scale bar 2.5 mm), E11.5 (B, scale bar 4 mm) and E12.5 (C, scale bar 5 mm). (D) Body weights of control and dko animals at E10.5 (n≥9), E11.5 (n≥22) and E12.5 (n≥11). (E) Scanning electron microscopy pictures of control and dko genital ridges at E11.5 (scale bar 200 µm). A pink overlay marks the genital ridges. (F) FACS analysis was used to determine the number of SF1^+^ somatic progenitor cells in genital ridges of control and dko animals at E10.5 (n≥9), E11.5 (n≥12), E12.5 (n≥13) and E13.5 (n≥6). (G) Double IF using the gonadal progenitor marker GATA4 (red) along with proliferating marker Ki67 (green) in control and dko genital ridges at E11.5. Quantification of GATA4^+^/Ki67^+^ cells in XX and XY genital ridges of control and dko animals at E10.5 (H) and E11.5 (I) revealed a significant reduction in the proliferation rates of somatic gonadal precursors at E10.5 and E11.5 in dko genital ridges. Values are expressed as means ± SEM, *p<0.05, **p<0.01, ***p<0.001 vs control. Scale bar: 100 µm.

Since IGFs stimulate both cell proliferation and differentiation, we investigated whether genital ridge development, its cellular composition and the number of multipotent somatic progenitors were affected in dko embryos prior to (E10.5) and around the time (E11.5–E12.5) of sex determination. We found that the overall body weight and embryonic growth appeared unaffected in dko embryos at E10.5, but began to diverge significantly at E11.5 with a 23% reduction, which then increase slightly to 27% at E12.5 (Figure 5A–5D). At all these stages (E10.5–E12.5), developmental processes such as tail somite formation and limb development were not delayed, suggesting that embryonic developmental processes were not affected despite growth retardation. Similarly, dko genital ridges were normally present at these developmental stages and their cellular composition appeared unaffected with the presence of gonocytes (OCT4^+^ cells) and somatic progenitors (GATA4^+^ cells) in both XX and XY dko genital ridges at E10.5 and E11.5 (Figure S2). Analysis of the urogenital anatomy by scanning electron microscopy at E11.5 did not reveal clear differences in the size and overall shape of XY dko genital ridges compared to controls (Figure 5E). However, by taking advantage of a transgene expressing eGFP under the control of the mouse *Sf1* promoter (*Sf1:eGFP*; [38]), we found by FACS that the number of SF1^+^ somatic progenitor cells was reduced by 42% (p = 0.0003) and 39% (p = 0.011) in dko genital ridges at E10.5 and E11.5, respectively (Figure 5F). As evidenced by double anti-GATA4/anti-Ki67 immunofluorescence, we found that the proliferation rates of gonadal progenitor cells (GATA4^+^) were significantly reduced at E10.5 and E11.5 in dko genital ridges compared to controls (Figure 5G–5I). In contrast, apoptosis rates did not differ between control and dko genital ridges at any time points examined (data not shown). A reduction in cell proliferation was also observed in other tissues such as the adjacent mesonephros, the somites and the heart suggesting that this effect is not specific to progenitor cells of the AGP but instead represents a more global effect of insulin/IGF signaling ablation (Figure S3).

### Decreased *Sf1* gene expression in the AGP and absence of adrenal glands in embryos lacking insulin/IGF signaling

**Figure 6:**
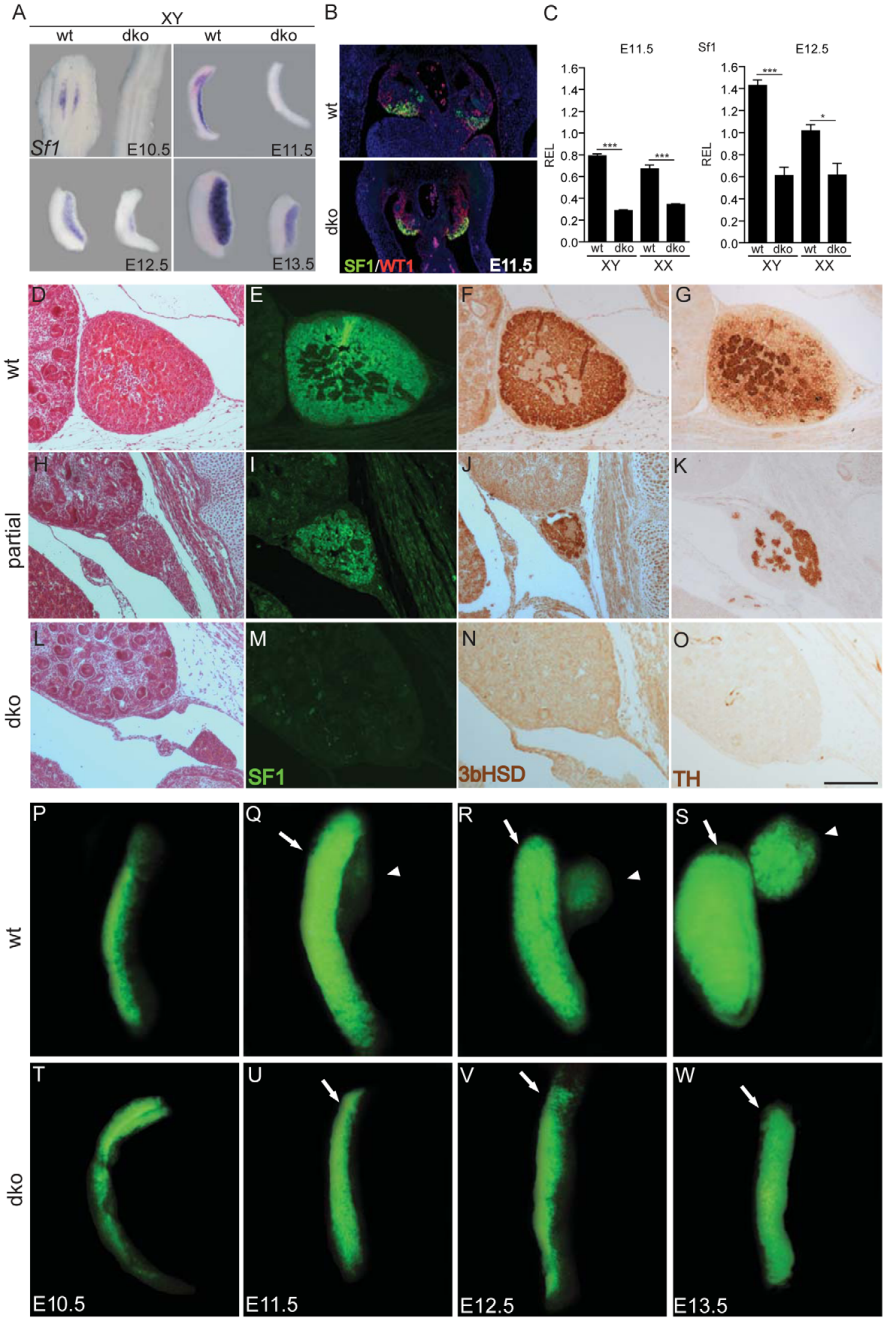
Absence of *Insr* and *Igf1r* negatively affects *SF1* gene expression and causes adrenal agenesis. Whole mount *in situ* hybridization on E10.5–E13.5 genital ridges (A), double IF with anti-SF1 (green) and anti-WT1 (red) at E11.5 (B) and qRT-PCR on genital ridges/mesonephroi at E11.5 and 12.5 (C) revealed that *Sf-1* is expressed at reduced levels both in XX and XY dko embryos during sex determination. Haematoxylin and eosin staining (D,H,L) as well as immunostaining for the adrenocortical markers SF1 (E,I,M) and 3βHSD (F,J,N) and chromaffin cell marker tyrosine hydroxylase, TH (G,K,O) from control (D–G) and dko (H–O) XY embryos at E16.5 revealed that adrenal glands are either massively reduced in size (H–K, 3 out of 27 embryos) or absent (L–O, 24 out of 27 embryos) in dko mutants. Fluorescence photomicrographs of XY genital ridges at E10.5 (P,T), E11.5 (Q,U), E12.5 (R,V) and E13.5 (S,W) from control (P–S) and dko (T–W) embryos expressing the *Sf1;eGFP* transgene. Note the absence of adrenal development (arrowhead) and gonadal primordium differentiation into testis (arrows) in dko animals. REL, relative expression levels. Values are expressed as means ± SEM, p*<0.05, ***p<0.001 vs control. Scale bar: 100 µm.

SF1 is a crucial determinant for the development and differentiation of the AGP. In fact, the onset of adrenal development has been reported to be more sensitive than gonadal development to *Sf1* gene dosage and requires a higher SF1 threshold [16], [39]. Interestingly, we found a significant reduction in *Sf1* transcript levels in mutant urogenital ridges between E10.5 and E12.5 irrespective of the genetic sex (Figure 6A–6C). We therefore investigated whether adrenal development was affected in embryos lacking both *Insr* and *Igf1r*. Examination of transverse abdominal sections by hematoxylin and eosin (H&E) staining revealed the absence of adrenal glands in dko embryos at E16.5 (compare Figure 6D with 6L). This was confirmed by the lack of staining for adrenocortical markers SF1 and 3β-hydroxysteroid dehydrogenase, 3β-HSD, and the chromaffin cell precursor marker tyrosine hydroxylase (TH) at E16.5 (compare Figure 6E–6G with 6M–6O). Among the large set of dko mice analyzed for the presence of adrenal structures at E16.5, 3 out of 27 embryos, all originating from the same litter, displayed a tiny adrenal structure expressing both steroidogenic (SF1, 3β-HSD; Figure 6I, 6J) and chromaffin cell markers (TH; Figure 6K). This suggested first that insulin/IGF signaling is required for adrenal cell specification but not for adrenocortical differentiation and/or function, and second that the genetic background affect the severity of the phenotype. Based on this striking observation, we next examined the early stages of adrenal development in dko embryos bearing a *Sf1;eGFP* transgene. Whereas adrenal and gonadal primordia are apparent in control embryos (arrowhead and arrow respectively in Figure 6P–6S), we observed a lack of GFP fluorescence specifically at the expected position of the adrenal primordium in dko embryos (Figure 6T–6W). These studies indicate that the insulin/IGF signaling pathway is indeed required for adrenal primordium specification, possibly by modulating *Sf1* gene expression.

### Ablation of *Insr* and *Igf1r* causes extensive alterations in testicular and ovarian transcription at E11.5

**Figure 7:**
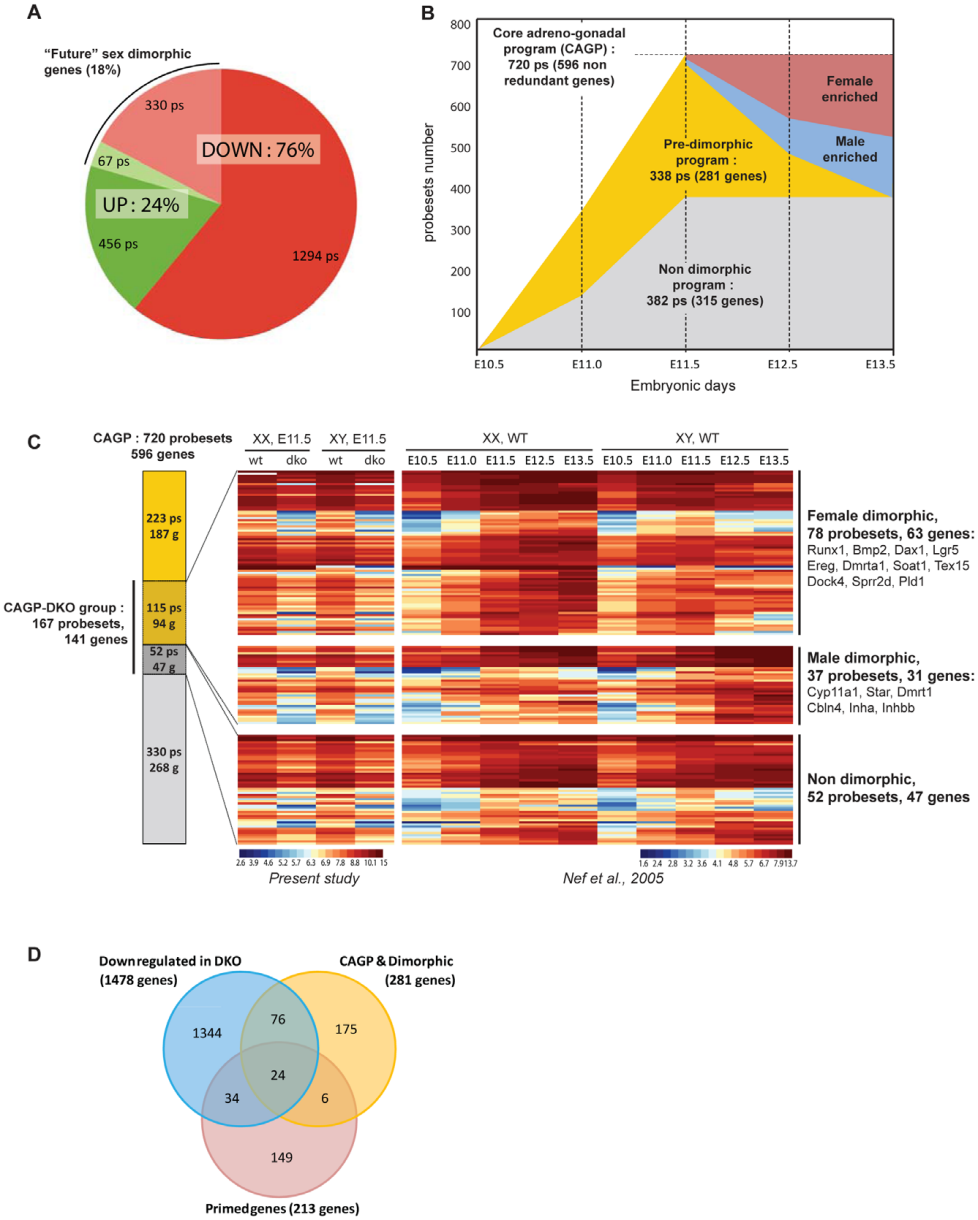
Ablation of *Insr* and *Igf1r* strongly affects the Core Adreno-Gonadal Program (CAGP), a group of genes responsible for the development of the bipotential gonad at the onset of sex determination. (A) Repartition of the probesets (ps) exhibiting variable expression profiles in dko gonadal Sf1^+^ cells compared to control at E11.5. 18% of these genes are “future” dimorphic genes; alterations in their expression in mutant Sf1^+^ cells at E11.5 precede the establishment of their known dimorphic expression pattern after E11.5. (B) Graphic representation of the upregulated genes in wild-type mouse embryonic gonads between E10.5 and E11.5 and their fate after E11.5. A total of 596 genes are upregulated in both female and male gonads between E10.5 and E11.5. This genetic program defines the Core Adreno-Gonadal Program (CAGP) and includes 281 “future” dimorphic genes (pre-dimorphic program). (C) Overlap of the CAGP and the list of down-regulated probesets (ps) and genes (g) in mutant Sf1^+^ cells at E11.5. In the mutant embryonic gonad, 141 genes out of the 596 genes of the CAGP are downregulated. The majority of these repressed genes are “future” dimorphic genes, with 63 female- and 31 male-specific genes. (D) Venn diagram representation of the overlap between genes from the dimorphic CAGP group, the primed genes group [41] and the set of genes downregulated in dko somatic progenitors.

To obtain a global view of the molecular changes associated with the ablation of insulin/IGF signaling, we performed a genome-wide gene expression analysis using Affymetrix microarrays on isolated SF1^+^ cells from XX and XY control and mutant gonads at E11.5. We chose this precise developmental stage as it corresponds to the peak of *Sry* expression and the initiation of both the testicular and ovarian genetic programs [10], [40]. We found that among the 2147 probesets affected in mutant SF1^+^ cells, 76% were down-regulated in the mutant gonads (Figure 7A, Figure S4A), revealing a strong negative impact on the transcription of genes associated with metabolic processes and cell cycle (for additional information see Figure S5 and Table S1). These changes may account for the reduced metabolism and proliferation observed in mutant somatic progenitor cells. In addition, the gene ontology analysis identified other down-regulated genes that are associated with sex determination, gonad development or steroid hormone synthesis. Strikingly, we found that 18% of the genes affected in dko gonads (350 annotated genes/397 probesets) are expressed in a sex-specific manner in SF1^+^ cells during testicular and ovarian development [10]. In other words, we identified several hundred genes exhibiting an altered expression profile in E11.5 dko gonads prior to the establishment of their sexually dimorphic pattern at later stages (i.e. E12.5 and E13.5; Figure 7A, Figure S4B). This included embryonic testis-specific genes such as *Atrx*, *Cyp26b1*, *Cbln4*, *Cyp11a1* and *Dmrt1* (Figure S6 and data not shown), as well as embryonic ovary-specific genes or female dimorphic genes such as *Bmp2*, *Cdkn1a*, *Cdkn1b*, *Runx1*, *Dax1*, and *Dmrta1* (Figure S7 and data not shown). All these genes were expressed at lower levels in mutant SF1^+^ cells regardless of genetic sex. This analysis suggested firstly that testicular and ovarian programs are initiated in the developing bipotential gonads in a gonadal sex-independent manner prior to E11.5, and secondly that initiation of these programs relies, at least partially, on insulin/IGF1 signaling.

### Alteration of a core adrenogonadal program (CAGP) in gonads lacking insulin/IGF signaling

In order to better characterize the sex-independent transcriptional program established in bipotential genital ridges prior to sex determination, and to investigate the role of insulin/IGF signaling in its initiation, we explored in more detail the expression profiles of genes enhanced and expressed in SF1^+^ somatic cell progenitors between E10.5 and E11.5. For this purpose, we carefully reexamined the transcriptome of SF1^+^ cells at E10.5, E11.0 and E11.5 in wild-type mouse embryonic gonads [10]. During this developmental period, 596 genes (720 probesets) were upregulated (fold change ≥1.5) both in XY and XX embryonic gonads and constitute what we call the Core Adreno-Gonadal Program (CAGP - Figure 7B). Interestingly, half of these CAGP genes (281 genes, 338 probesets) later exhibited a sexually dimorphic expression pattern, indicating their association with the testicular or ovarian genetic programs.

Strikingly, our transcriptomic analysis comparing control and double mutant SF1^+^ cells at E11.5 revealed that the absence of insulin/IGF signaling affects the expression of more than 23% of the CAGP (141 out of 596 genes; Figure 7C), and 33% of the subset of CAGP genes with subsequent sexually dimorphic expression patterns (94 out of 281 genes). It includes genes such as *Runx1*, *Bmp2* and *Dax1* in mutant XX embryos and *Cyp11a1*, *Dmrt1* and *Cbln4* in mutant XY embryos. Using our SF1-GFP expression data [10], Jameson et al. [41] identified a group of 213 genes, named “primed genes” that are initially expressed at identical levels both in XX and XY somatic progenitors prior to sex determination but then become sexually dimorphic when these cells adopt either a male of female fate. This group of 213 genes is different from the CAGP described above, despite a small overlap, but was affected to a similar extent in the absence of Insulin/IGF signaling: expression levels of 27% of primed genes were reduced in the dko gonads (Figure 7D). All together, these data clearly emphasize the essential role played by insulin family growth factors in establishing both the male and female programs in XX and XY somatic progenitors prior to sex determination.

**Figure 8:**
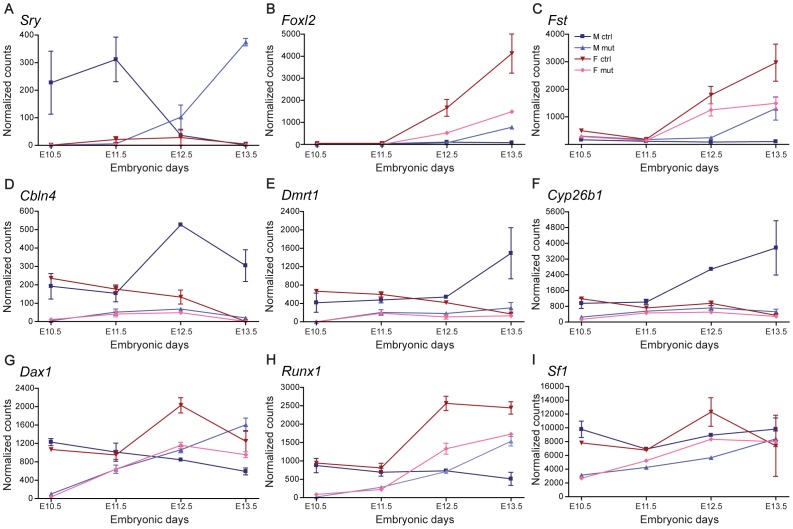
Expression profiles of key genes in adrenogonadal development and differentiation as determined by NanoString Multiplex Assays. Total RNAs were isolated from SF1^+^ cells isolated from XX and XY control or dko gonads between E10.5 and E13.5 control female mice (red); control male mice (blue); dko female mice (light red); dko male mice (light blue). Normalized counts for genes such as (A) *Sry*, (B) *Foxl2*, (C) *Follistatin* (*Fst*), (D) *Cerebellin 4* (*Cbln4*), (E) *Dmrt1*, (F) *Cyp26b1*, (G) *Dax1*, (H) *Runx1* and (I) *Sf1* were normalized with the geometric mean of the 6 reference genes. Bars represent the standard deviation.

To validate these results and confirm that the expression of significant fractions of CAGP and primed genes were affected as early as E10.5 in mutant progenitor cells, we developed an assay based on the NanoString Ncounter gene expression system, which captures and counts individual mRNA transcripts without reverse transcription of RNA or any other enzymatic step [42]. We measured the expression profiles of a set of 65 genes in SF1^+^ cells isolated from XX and XY control or dko gonads between E10.5 and E13.5 (Figure 8, Figure S8 and Table S2). This set of genes included classical genes involved in adrenogonadal development and sex determination as well as a selection of CAGP and primed genes. Analysis of the sources of variation (ANOVA) indicated that the most significant factors influencing gene expression variation were the time (developmental stages) followed by genotype (control vs dko) and sex (Figure S8A). As expected, genes implicated in the testicular program were not upregulated in SF1^+^ cells from XY dko gonads (Figure 8D–8F; Figure S8B) with the notable exception of *Sry* whose peak of expression was delayed by 2 days (Figure 8A). Similarly, we confirmed that the expression profile of numerous ovarian genes such as *Foxl2*, *Fst* and *Lef1* were delayed and reduced in SF1^+^ cells from both XX and XY dko gonads, compared to XX control ovaries (Figure 8B, 8C and Fig S8B). Finally, we also confirmed that CAGP and primed genes were indeed expressed both in XX and XY SF1^+^ somatic progenitors from E10.5 to E11.5 but were reduced or absent in mutant progenitor cells at the same stages. Several representative examples such as *Cbln4*, *Dmrt1*, *Cyp26b1*, *Dax1* and *Runx1* are shown in Figure 8. Also of particular interest is *Sf1* whose expression was downregulated by ∼67% and ∼38% at E10.5 and E11.5, respectively.

Overall, we showed that a complex dynamic transcriptional program, entitled the Core Adreno-Gonadal Program (CAGP), is initiated in the bipotential gonadal primordium prior to sex determination and is associated with testicular and ovarian differentiation as well as adrenal specification. The significant alteration in CAGP and primed gene expression in somatic cells of the *Insr;Igf1r* mutant AGP prior to sex determination may explain the incapacity of this primordium to specify not only the adrenal gland but also to develop into either ovaries or testes in a timely fashion.

## Discussion

Both the gonads and the adrenal cortex originate from a common structure referred to as the adreno-genital primordium (AGP). Although insulin-like growth factors (IGFs) provide essential signals for the control of embryonic development, their implications in mediating AGP development and differentiation are poorly understood. Using *in vivo* models, we demonstrate that the insulin receptor tyrosine kinase genes *Insr* and *Igf1r* are required for adrenal development and gonadal differentiation. Mouse embryos lacking *Insr* and *Igf1r* exhibit reduced proliferation rates of somatic progenitor cells in both XX and XY gonads prior to sex determination together with complete agenesis of the adrenal gland and absence of testis development due to a reduction in *Sf1* gene expression and a failure of *Sry* upregulation. In addition, we observed a delay in ovarian differentiation and germ cell entry into meiosis suggesting that, irrespective of the genetic sex, gonads lacking insulin signaling remain in an undifferentiated state with no clear activation of either testicular or ovarian genetic programs for several days. Expression analysis of SF1^+^ somatic cells during sex determination reveals that significant fractions of the testicular and ovarian genetic programs are prematurely altered, which could explain both adrenal agenesis and the incapacity of mutant gonads to develop into either ovaries or testes at the time of sex determination.

Surprisingly, we found that loss of *Irr*, one of the three members of the insulin receptor tyrosine kinase family comprising INSR, IGF1R and IRR, is not required for sex reversal as it was reported in our intitial publication [21]. We hypothesize that variations in the genetic background of the double *Insr*;*Igf1r* and triple *Insr*;*Igf1r;Irr* knockout colonies might explain the differences in phenotype. The two studies used different genetically modified mouse lines; while the former used constitutive ko alleles for *Insr*, *Igf1r* and *Irr*, this study involved no less than five different transgenes (*Insr* and *Igf1r* floxed alleles, *Sf1-GFP* tg, *Gdf9:Cre* tg, *Ngn3:Cre* tg) none of them being used in the previous study. Overall, the results remain rather similar with an essential contribution to AGP development and differentiation by IGF1R, followed by INSR, whereas IRR contribution to this developmental aspect is, if at all, minimal.

Little is known about the factors regulating AGP development although several genes encoding transcription factors, including *Sf1*, *Wt1*, *M33*, *Cited2*, *Pbx1*, *Odd1* and *Lhx9*, have been implicated (for a review see [43]). These genes act either in a hierarchical manner or via protein-protein interactions to regulate target genes essential for adreno-gonadal development and function. Targeted deletions of these genes usually manifest themselves in defects in the development of both adrenal and gonadal tissues [12]. Interestingly, the concomitant ablation of *Insr* and *Igf1r* is the first example of null mutations in growth factor receptors, as opposed to transcription factors, that lead to similar defects. We believe that the reduced number of somatic progenitors observed in dko gonads prior to sex determination (i.e. −39% at E11.5) is not severe enough to cause sex reversal by itself. Studies of XX–XY chimaeric mice have indicated that as little as 20% of *Sry*-expressing Sertoli precursor cells direct testicular development, whereas ovarian differentiation will occur when less than 20% of XY cells are present [44]. The absence of insulin/IGF signaling affects not only the proliferation of SF1^+^ cells prior to sex determination but also the expression of significant proportions of two groups of genes: the primed genes, and the core adrenogonadal program (CAGP). The coordination of cell proliferation with cell fate decisions underpins any developmental process. We believe that the reduced proliferation rate observed in dko embryos may have a profound impact on chromatin remodeling in SF1^+^ somatic progenitor cells prior to sex determination, and consequently affects both the CAGP and adreno-gonadal developmental process [45]. The CAGP is a rather large genetic program of ∼600 genes that are upregulated in both XX and XY SF1^+^ progenitor cells prior to sex determination. Approximately half of these genes exhibit a sexually dimorphic expression pattern at later stages, suggesting that they are associated with the differentiation and/or function of adrenal, testicular and ovarian tissues. In fact, these findings suggest that early gonadal progenitor cells in both XX and XY gonads express at low levels a large set of genes associated with the testicular and ovarian genetic programs, thus establishing a bipotential state for the uncommitted gonad. During sex determination, these multipotent somatic cells will then upregulate gene expression associated with the adopted fate, and repress markers of the other fates [41]. In mice lacking insulin/IGF signaling, we observed that a significant fraction of both primed genes (27% or 58 genes) and dimorphic CAGP genes (33% or 94 genes) were affected as early as E10.5 in somatic progenitors of the developing gonad. This may explain the incapacity of double mutant gonads to differentiate into either a testis or an ovary. To our knowledge, this is the first time that an uncommitted gonad remains in an undifferentiated state for several days after sex determination should occur, until the ovarian differentiation program finally takes over by E16.5.

Recently, it has been shown that supporting cell progenitors of the gonad are “lineage primed” by expressing genes characteristic of both the ovarian and testicular programs, but that the ovarian program is over-represented and predominant [41]. The over-represented ovarian program observed in these somatic progenitor indicates a closer relationship to the female fate. It may also explains why the female fate is the “default” state and lead to ovarian differentiation in the absence of *Sry*, and why the ovarian program ultimately takes over in XX and XY dko gonads although effective differentiation is delayed for several days. At E11.5, both the ovarian and testicular genetic programs are significantly affected in mutant progenitor cells. Thus, dko gonads remain in an undifferentiated state for several days until the ovarian program gets finally activated to a sufficient threshold that ultimately leads to ovarian differentiation in the absence of an intervention from *Sry*.

Nevertheless, it remains unclear which genes affected in *Insr;Igf1r* mutant gonads impair the capacity of the mutant AGP to specify adrenal gland, and promote testicular or ovarian differentiation. Expression analysis revealed that transcript levels for transcription factor genes essential for adreno-gonadal development and function, such as *Wt1*, *Lhx9* and *Sf1*, were reduced ∼2 fold prior to sex determination (Figure 6A, 6C and Figure S8B). In addition, 134 genes from the dimorphic CAGP group and/or the primed gene group were significantly affected in mutant genital ridges prior to and/or at the time of sex determination. Since gene dosage of *Sf1* is critical for adreno-gonadal development both in humans and mice [39], [46], the reduced expression of this transcription factor may explain a significant part of the phenotype observed in *Insr;Igf1r* mutant animals. Mice deficient in *Sf1* undergo early adrenal and gonadal development but then regress by E12.5 [13]. Adrenal gland development is more sensitive to *Sf1* dosage than that of the gonads [39]. Similarly to *Insr;Igf1r* mutant embryos, *Sf1* haploinsufficient mice exhibit adrenal insufficiency due to a severe reduction of adrenocortical precursors within the AGP, but show no change in the number of gonadal precursors [39]. However, it remains unclear how IGFs regulate *Sf1* gene expression in somatic progenitor cells and whether the phenotype observed in dko mutant mice could be attributed to a cell-specific or a more indirect, global effect. One hypothesis is that IGFs regulate directly *Sf1* gene transcription or its activity through the PI3K/AKT and/or the MAPK signaling pathways by regulating its phosphorylation status or by influencing other transcription factors [47]. However, to our knowledge, no study has ever reported that IGFs regulate directly SF1 expression/activity in steroidogenic cells of the adrenal gland, testis and ovary. Alternatively, IGFs may affect *Sf1* gene transcription indirectly, together with a large set of genes including genes related to gonadal differentiation (i.e. primed genes and CAGP genes) as well as numerous other genes associated with cell cycle regulation, metabolism, steroid hormone synthesis, nervous system development, skeletal system development or cartilage development. This global effect on gene transcription may simply reflect the fact that mutant SF1^+^ supporting progenitor cells are delayed in their progression from a quiescent and pluripotent state toward the differentiation cascade.

### Insulin/IGF signaling and the male pathway

Initiation of the testicular pathway requires a threshold level and the correct timing of *Sry* and *Sox9* expression (for review see [48]). SF1 is an essential transcription factor known to promote Sertoli cell differentiation and the testicular pathway by participating in *Sry* activation and the initiation, upregulation and maintenance of *Sox9* transcription in Sertoli cell precursors [48]. We found that *Sry* expression was drastically reduced and delayed in *Insr;Igf1r* double ko animals, and was correlated with the lack of upregulation of key testis genes such as *Sox9*, *Fgf9*, and *Ptgds*, and the absence of Sertoli cells, Leydig cells and overall testis formation. Interestingly, a few SOX9^+^ cells were found in E12.5 XY dko gonads. These were absent at later stages suggesting that, in addition to *Sox9* activation, maintenance of *Sox9* expression was also impaired in mutant XY gonads. Recent studies demonstrated that the first event occurring immediately downstream of the onset of SRY expression is the accumulation of glycogen within the precursors of Sertoli cells [49]. This energy storage is critical since disruption of glycogen synthesis and accumulation results in the failure of *Sox9* upregulation, testis cord formation and overall testis development. Interestingly, glycogen storage within pre-Sertoli cells appears to be dependent on the activation of the PI3K-AKT pathway, which is known to be activated by both insulin and IGFs [20]. Both expression profiling and Affymetrix analyses showed that genes coding for enzymes involved in the glycogen synthesis pathway, such as *hexokinase 2* (*Hxk2*), *phosphoglucomutase* (*Pgm*) and g*lycogenin* (*Gyg*), were downregulated in dko SF1^+^ cells at E11.5 (Figure S9). In addition, qRT-PCR performed with RNAs isolated from genital ridges at E11.5 showed that *glycogen synthase* (*GlycoS*) was also down regulated in dko embryos.

### Insulin/IGF signaling, ovarian differentiation, and meiosis

Absence of insulin/IGF signaling led to a delay in the ovarian program of development, which was reflected at the molecular level by an absence or reduced expression of numerous genes involved in ovarian development. This included FOXL2, an ovarian determining factor, as well as members of the *Wnt4* signaling pathway such as *Wnt4*, its downstream gene *Fst* and the nuclear mediator of canonical WNT signaling, *Lef1*. Combined with the failure to initiate the testicular program, *Insr;Igf1r* mutant gonads remained in an undifferentiated state for several days, until E16.5 when the ovarian program was activated in both XX and XY embryos. This delay in differentiation is apparent for both germ and somatic cell lineages in the gonad. Ordinarily, following the initiation of the ovarian differentiation program in the somatic compartment of XX gonads, germ cells enter into prophase of meiosis I around E13.5 [50] and upregulate meiotic proteins including the synaptonemal complex 3 (SCP3). In the fetal ovary, germ cell entry into meiosis is induced by retinoic acid. In the developing testes however, expression of the P450 enzyme CYP26B1 in Sertoli cells, which degrades retinoic acid [7], [8], and secretion of FGF9 that directly suppress meiosis, act to maintain pluripotency [51]. Although germ cells were present normally in both XX and XY double mutant gonads, we observed an almost complete absence of SCP3-positive cells at E12.5 and E13.5 (Figure 4). Besides the numerous other genes that are affected in the double mutant gonads, several factors regulating retinoic acid (RA) metabolism and the correct specification of the germ cell lineage exhibited a marked decrease in mutant SF1^+^ cells. These included not only *Fgf9* and the retinoic acid degrading enzyme *Cyp26b1*, but also the aldehyde dehydrogenases *Aldh1a1* and *Aldh1a7* as well as the alcohol dehydrogenase *Adh1* (Figure S6). These latter enzymes catalyze retinol oxidation, the rate-limiting step in the conversion of retinol to retinoic acid. Consistent with the reduction of *Aldh1a1* and *Adh*, we observed a similar reduction in several RA-regulated genes such as *Runx1*, *Pbx1*, *Bmp2* and *Tgfb2* (data not shown). We hypothesize that alteration of both meiosis-suppressing factors (e.g. *Fgf9, Cyp26b1*) and meiosis-promoting factors such as key synthesizers of retinoic acid in the mesonephros (e.g. *Aldh1a1*, *Aldh1a7*, *Adh*) perturbs the initiation of meiosis and germ cell fate in *Insr;Igf1r* mutant animals.

### What are the signaling pathway(s) mediating IGF actions within the developing AGP?

The insulin/IGF family of growth factors acts mainly through INSR and IGF1R to activate two major signaling pathways: the mitogen-activated protein kinase (MAPK) pathway and the phosphoinositide 3-kinase (PI3K)/Akt pathway [20]. The MAPK pathways, including EKR1/2, P38 and JNK, regulate cell proliferation, differentiation and apoptosis, while increased phosphatidyl inositol 3,4,5 triphosphate (PIP3) activates PKB/AKT to prevent apoptosis and to stimulate cellular proliferation and glucose transport. Recently, MAPK pathways have been implicated in testis development: mutations in the *MAP3K1* gene cause 46,XY disorders of sex development with partial or complete gonadal dysgenesis [52]. In addition, loss of function of the *Map3k4* gene in mice led to XY gonadal sex reversal [30]. Analysis of mutant gonads revealed a dramatic reduction of *Sry* and *Sox9* expression and a subsequent growth deficit and absence of mesonephric cell migration. Expression analysis of genes coding for proteins involved in insulin/IGF signaling, in particular both the MAPK and PI3K/Akt signaling pathways, revealed that most of these genes were not affected in the double knockout gonads, with the notable exception of *Map3k1*, *Map2k7*, *Jnk3/Mapk10*, *Gadd45γ*, *p38β*, *p38γ* and *p38δ* (Figure S10). However, it is expected that many physiological functions of IGFs in developing gonads are mediated through post-translational modifications, such as phosphorylation, of downstream signaling effectors. A major future task will be to define the signaling pathways that mediate these proliferative activities, and that allow these growth factors to specify the adrenal primordium and promote testicular and ovarian differentiation.

In conclusion, this study demonstrates the essential role played by the insulin/IGF signaling pathway in mediating different aspects of adrenogonadal development, such as adrenal specification, testicular differentiation and ovarian development. It also sheds light on a crucial, but so far underestimated, signaling pathway underlying sex determination in mice and potentially disorders of sexual development in humans.

## Materials and Methods

### Reagents, antibodies, and primers

All the reagents, antibodies, plasmids and primers used in this study are described in Tables S3 and S4.

### Animals

Insr^flox^(*Insr^fx/fx^*), Igf1r^flox^ (*Igf1r^fx/fx^*), Sf1-eGFP (*Sf1:eGFP*) Ngn3-Cre (*Ngn3:Cre*) and Gdf9-Cre (*Gdf9:Cre*) transgenic mice were provided by R. Kahn, A. Efstratiadis, K.L. Parker, P.L. Herrera and A.J. Cooney respectively and were genotyped at weaning (P21) from tail biopsies by classic PCR as described [22], [24], [26], [38], [53]. To generate constitutive mutants for both *Insr* and *Igf1r, Insr^fx/fx^;Igf1r^fx/fx^;Gdf9:Cre* XX mice were mated with *Insr^fx/fx^;Igf1r^fx/fx^;Ngn3:Cre* XY mice. The genotype of gametes produced by both transgenic lines were *Insr* ^Δ^;*Igf1r* ^Δ^ and the subsequent matings resulted in 100% *Insr* ^Δ/Δ^;*Igf1r* ^Δ/Δ^ progeny, hereafter referred as “dko”. The genotype of control mice was *Insr^fx/fx^;Igf1r^fx/fx^*. To specifically label SF1 expressing cells *in vivo*, the *Sf1:eGFP* transgene [38] was intercrossed with the above mentioned mice to generate *Insr^fx/fx^*;*Igf1r^fx/fx^*;*Sf1:eGFP, Insr^fx/fx^*;*Igf1r^fx/fx^*;*Gdf9:Cre*;*Sf1:eGFP* and *Insr^fx/fx^*;*Igf1r^fx/fx^*;*Ngn3:Cre*;*Sf1:eGFP* transgenic animals. Embryos were collected from timed matings and staged by designating noon of the day on which the mating plug was detected as E0.5. Accurate staging of embryos between 10.5 and 12.5 dpc was performed by counting the tail somites (ts). Embryos at 8 ts (±2 ts) were considered as E10.5, 19 ts (±2 ts) as E11.5 and 29 ts (±3 ts) as E12.5. Routine sexing of the embryos was determined by *Sry* PCR [10]. Due to the large number of different transgenes involved in these breedings, the genetic background of dko and control embryos is mixed, although mostly composed of 129 and BL/6 strains. Animals were housed and cared according to the ethical guidelines of the Direction Générale de la Santé of the Canton de Genève (experimentation ID: 1061/3840/1).

### Morphology, histology, and immunohistochemistry

Following timed matings, embryos were fixed overnight at 4°C in either 4% paraformaldehyde (PFA) or Bouin's fixative, dehydrated in an ethanol series and embedded in paraffin. Five µm-sections were stained with hematoxylin and eosin (H&E) or processed for section immunohistochemistry (IHC) and immunofluorescence (IF). Section IHC and IF was performed as described [5].

### Western blots

Total protein from E11.5 control and dko embryos were mechanically homogenized in ice-cold RIPA buffer. Lysates were cleared by centrifugation and protein content was measured using a BCA protein assay kit. Samples containing 10 µg of total protein were resolved by 10% SDS-PAGE, transferred to nitrocellulose membranes and following staining with antibodies were detected using Lumigen TMA-6 according to manufacturer's instructions.

### Isolation of purified Sf1:GFP positive cells and RNA extraction

Adult females were time-mated and checked for the presence of vaginal plugs the next morning (E0.5). On the relevant days of gestation (i.e. E10.5, E11.0, E11.5, E12.5 and E13.5), pregnant females were sacrificed. XX and XY urogenital ridges from dko and control embryos bearing the *EGFP* transgene were dissected and digested with trypsin/EDTA. eGFP-positive cells were sorted using a FACS Vantage SE as described [10]. The levels of GFP fluorescence of SF1+ cells were comparable and were not affected by the genetic sex and the genotype of developing embryos (see Figure S11). We only observed a small and regular increase in the levels of GFP fluorescence based on the developmental stage that did not affect the cell sorting process. RNA was extracted using RNeasy microkit from Qiagen according to the manufacturer's protocol and stored at −80°C until needed.

### Probe synthesis and microarrays

For each of the 4 genotypes (XX and XY control, XX and XY dko), three independent sets of 150 ng of total RNA were isolated and used as a template for probe generation as described [54].

#### Raw data production and preprocessing

Microarrays were scanned with a GeneChip scanner 3000 7G (Affymetrix). Raw image files (DAT) as well as feature-level data files (CEL) were generated using the GeneChip Operating System (GCOS 1.4, Affymetrix). CEL files were uploaded into the AMEN v.1.3.4 software [55]; http://sourceforge.net/project/AMEN) and submitted to the Robust Multi-array Average (RMA) procedure allowing for summarization of probeset intensities, background correction and data normalization [56]. The data were quality controlled by scatter and box plots as implemented in AMEN software 1.3.4 as well as a Kullback-Leibler distance matrix [57]; Figure S12). CEL and RMA-normalized expression files are available at the EBI ArrayExpress public data repository (accession no. E-MTAB-1156; http://www.ebi.ac.uk/microarray-as/aer/index.html). CEL data files corresponding to mouse XX and XY Sf1^+^ cells from E10.5 to E13.5 [10] were downloaded from the EBI ArrayExpress repository (accession no. E-MEXP-454) and normalized with the AMEN software using the RMA procedure.

#### Probeset and target sequence annotation

The Affymetrix annotation file (CSV format) corresponding to MOE-230 2.0 GeneChip arrays (http://www.affymetrix.com/support/index.affx, release 30) was uploaded in the AMEN v.1.3.4 software and further completed with HomoloGene (ftp://ftp.ncbi.nih.gov/pub/HomoloGene/current/homologene.data, 08/08/09, build 64) and Gene Ontology (GO) (ftp://ftp.ncbi.nih.gov/gene/DATA/gene2go.gz, 20/09/10 release) term identifiers (IDs) [58].

#### Statistical data analysis

To identify differentially expressed genes/transcripts, we performed a pairwise comparison analysis using the AMEN v.1.3.4 software. The four experimental genotypes (XX and XY wild-type, XX and XY double mutant) were compared by considering only one biological variation (sex or genetic modification). For each of the four comparisons, we retained as differentially expressed those probesets with an intensity value superior to the global median (6.295062) in at least one of the two compared conditions, which exhibited a fold change of 1.5 or above (FC≥1.5) between the averaged values of the two selected conditions and which satisfied a Limma differential expression test (p-value*≤*1%, BH-adjusted; [59]).

An identical strategy was used to identify the genes up-regulated in XX or XY gonads between E10.5 and E11.5 using our previously published microarray dataset [10]; global median = 4.109341, Limma differential expression test with p-value*≤*1%, BH-adjusted). This group of genes, which was called Core Adreno-Gonadal Program, was then compared with the lists of genes down-regulated in mutant gonads, as well as ovarian and testicular dimorphic genes previously identified by us [10] and primed genes characterized by Jameson et al. [41]. In order to avoid duplicates, genes with multiple probesets exhibiting contradictory expression profiles were removed from the analysis.

#### Cluster analysis and functional data mining

Specific expression patterns were defined through K-mean clustering (Complete linkage) using the AMEN v.1.3.4 software. Biological mining of expression clusters was performed by searching for over- and under-represented functional annotation terms from the GeneOntology associated with non-redundant gene IDs (EntrezGene).

### Whole-mount *in situ* hybridization (WISH)

WISH was carried out as described [10]. Briefly, embryos were dissected in PBS, fixed overnight in 4% PFA at 4°C, washed in PBS, and then dehydrated in graded methanol solution and stored at −20°C in 100% methanol. Plasmids containing cDNAs of the relevant candidate genes were linearized and then used as templates to generate digoxigenin-labeled anti-sense riboprobes. Expression profiles were analyzed at E10.5, E11.5, E12.5, E13.5 and E16.5 using a minimum of three embryos of each sex and genotype at each stage per candidate gene.

### Quantitative RT–PCR (qRT–PCR)

Total RNAs from E11.5 (19±2 ts) and E12.5 genital ridges (together with mesonephroi) from XX or XY, control or dko embryos were extracted using the RNeasy microkit from Qiagen according to the manufacturer's protocol. For each RNA sample, 15 pairs of genital ridges from the same genotype and stage were pooled. For each condition, three independent pools of RNA were isolated, DNase-treated and converted to 1^st^ strand cDNA using SuperScript II Reverse Transcriptase following the manufacturer's instructions (Invitrogen Life Technologies). Real time PCR was carried out in optical 384-well plates and labeled by using the SYBR green master mix (Applied Biosystems), and the fluorescence was quantified with a Prism 7900 HT sequence detection system (Applied Biosystems). The expression of each gene was assayed in triplicate as previously described [54]. Primers used for qRT-PCR are listed in Table S3 and were designed using the software PRIMER EXPRESS (Applied Biosystems). The statistical significance of fold-changes was determined by a paired Student's *t*-test.

### NanoString Ncounter gene expression system

Total RNAs were isolated from purified SF1^+^ cells originating from XX and XY urogenital ridges at E10.5, E11.5, E12.5 and E13.5 from both dko and control embryos (19±2 ts) bearing a *Sf1:EGFP* transgene as described [10]. For each of the 16 conditions, 3 independent sets of total RNA (each originating from a pool of >6 embryos) were isolated to minimize the effects of biological variability. For each condition, 100 ng of total RNA was hybridized with multiplexed Nanostring probes and samples were processed according to the published procedure [42]. Barcodes were counted for 1150 fields of view per sample. Background correction was done by subtracting from the raw counts the mean+2 standard deviations of counts obtained with negative controls. Values <1 were fixed to 1. Positive controls were used as quality assessment: the ratio between the highest and the lowest positive controls average among samples was below 3. Then counts for target genes were normalized with the geometric mean of the 6 reference genes (*Gapdh*, *Tuba1b*, *Gusb*, *Eef1a1*, *Tbp* and *Rps9*) selected as the most stable using the geNorm algorithm [60].

### Statistical analysis

Results of a representative experiment are shown and are expressed as means ± SEM of *n* experiments. The nonparametric unpaired *t*-test was used for statistical analysis. Differences were considered statistically significant if *p* was <0.05.

## Supporting Information

Figure S1 Absence of testicular markers in XX dko gonads. The expression of key testicular markers was assessed in control and dko XX gonads at E12.5, E12.5 and E16.5 with double immunofluorescence using either the Sertoli cell marker SOX9 (A–F, in green) or the Leydig cell marker P450SCC (G–L, in green) along with E–cadherin (red).
(PDF)

Figure S2 Markers of both somatic progenitors and germ cells are normally present in dko gonads at E10.5 and E11.5. The presence of both somatic precursors and germ cells in control and dko gonads at E10.5 and E11.5 was assessed by immunofluorescence using either the somatic progenitor cell marker GATA4 (A,B, in red) or the germ cell marker OCT4 (C,D, in red). Gonadal tissues are delimited by dotted lines.
(PDF)

Figure S3 Reduced proliferation rates in somites, mesonephroi and heart of dko embryos at E10.5 and E11.5. (A) Cell proliferation in the developing somites, mesonephroi and heart was evaluated by immunofluorescence using the proliferating marker KI67 (green). In somites and heart, proliferating cells are positive for KI67 (green) and DAPI (blue), whereas in the mesonephros, proliferating cells are KI67^+^, DAPI^+^ but GATA4 negative (red). (B) Quantification of KI67^+^/DAPI^+^ revealed a significant reduction in the rate of proliferation of the cells composing the somites, the mesonephroi and heart in dko embryos both at E10.5 and E11.5. The proliferation rate was evaluated using three different embryos for each sex, stage and genotype and a minimum of three slides per embryo (n>9). Values are expressed as means ± SEM, p*<0.05, p**<0.01, ***p<0.001 vs control.
(PDF)

Figure S4 Representation of the 397 probesets being differentially expressed in dko SF1^+^ cells. (A) Graphic representations of the number of probesets found up- (green) or down-regulated (red) for each of the pairwise comparisons performed between the 4 analyzed samples. (B) Heatmap representation of the probesets exhibiting an altered signal in mutant SF1^+^ cells and a dimorphic expression pattern between male and female embryonic gonads. Female and male dimorphic genes were clustered into two groups according to the expression profiles described in Nef et al 2005 ([10], indicated on the right). A K-means clustering strategy was performed to identify probesets within these two groups that were up- and down-regulated in dko SF1^+^ cells (indicated on the left). The first four columns of the heatmap summarize the expression patterns of the 397 probesets within the control (WT) and double mutant (DKO) samples at embryonic day 11.5 (E11.5), whereas the last ten columns show the expression profiles of these probesets in female (XX) and male (XY) between E10.5 and E13.5. Expression levels are indicated by blue (low) or red (high) colouring.
(PDF)

Figure S5 Genes from known biological processes are affected in dko gonads. Selection of a set of Gene Ontology (GO) terms found enriched within the two groups of up- and down-regulated genes. Each cluster is matched with enriched GO terms from the ontologies “biological process”, “molecular function” and “cellular component”, and the numbers of genes (Entrezgene id) associated with a specific GO term and enriched in each cluster are given within rectangles as observed (left number) and as expected (right number). The color of the rectangle indicates overrepresentation (red) and underrepresentation (blue) as indicated in the scale bar.
(PDF)

Figure S6 Expression profiles of known male dimorphic genes in dko gonads. Probeset intensities between XX and XY control and dko mutant SF1^+^ cells at E11.5 were pairwise compared and submitted to an unpaired t-test to evaluate the significance of differences between samples, *p<0.05, **p<0.01, ***p<0.001 vs control. Graphs illustrating the expression profiles of the selected probesets in SF1^+^ cells at different times of gonad sex determination as determined in Nef et al 2005 ([10]). The peak expression level is set as 100%, and the expression levels at other time points are relative to peak levels (% peak). Bars represent the standard deviation.
(PDF)

Figure S7 Expression profiles of known female dimorphic genes within the dko gonads. Probeset intensities between XX and XY control and dko mutant SF1^+^ cells at E11.5 were pairwise compared and submitted to an unpaired t-test to evaluate the relevance of differences between samples, *p<0.05, **p<0.01, ***p<0.001 vs control. Graphs illustrating the expression profiles of the selected probesets in SF1^+^ cells at different times of gonad sex determination as determined in Nef et al 2005 ([10]). The peak expression level is set as 100%, and the expression levels at other time points are relative to peak levels (% peak). Bars represent the standard deviation.
(PDF)

Figure S8 Expression profiles of 65 genes as determined by NanoString Multiplex Assays. (A) Analysis of the sources of variation (ANOVA) revealed that the main sources of variations are genotype, sex and time. (B) The set of selected genes includes classical genes involved in the testicular program, the ovarian program, adrenogonadal development and insulin/IGF signaling as well as a selection of CAGP and primed genes. Total RNAs were isolated from SF1^+^ cells isolated from XX and XY control or dko gonads between E10.5 and E13.5 control female mice (red); control male mice (blue); dko female mice (light red); dko male mice (light blue). Bars represent the standard deviation.
(PDF)

Figure S9 Alteration of genes involved in the glycogen synthesis pathway. Pathway for conversion of glucose monomers to polymeric glycogen including relevant enzymes. Transcript levels for these enzymes were assessed in XY and XX control and dko gonads at E11.5 and E12.5 by Affymetrix analyses or by qRT-PCR. Values are expressed as means ± SEM, **p<0.01, ***p<0.001 vs control.
(PDF)

Figure S10 Alteration of genes involved in the MAPK pathway. Transcript levels were assessed in XY and XX control and dko gonads at E11.5 and E12.5 by Affymetrix analyses or by qRT-PCR. Values are expressed as means ± SEM, **p<0.01, ***p<0.001 vs control.
(PDF)

Figure S11 GFP fluorescence of SF1^+^ cells was not affected by the genetic sex and the genotype of developing embryos. Representative FACS graphs showing the typical and identical pattern of Sf1/eGFP^+^ cell distribution from control (A) or dko (B) XY genital ridges at E11.5. FL1H axis represents the levels of fluorescence (arbitrary units) while the x axis reflects the size of the cell. (C) Relative levels of Sf1/eGFP fluorescence were not affected by the sex and the genotype. For each condition tested, a mimimum of 6 embryos was analyzed. The nonparametric unpaired *t*-test was applied for statistical analysis. The abbreviation ns denotes non-significant changes (p>0.05).
(PDF)

Figure S12 Validation of the RMA normalization process. (A) Box plot representation of expression data before and after RMA normalization. Scale bar indicates Log2-transformed probeset intensities. WT = control; Mut = dko. (B) Correlation matrix for sample duplicates. High and low degrees of similarity between two samples are displayed in black and white, respectively. WT = control; Mut = dko.
(PDF)

Table S1 List of Affymetrix mouse 430.2 microarray probesets differentially expressed between control and dko gonads at E11.5. We provide for each probeset: the characteristics of the corresponding genes (probeset IDs, Entrez gene, homologene, GeneName, and expression group); the log2-transformed expression values and fold changes associated to each of the 4 experimental conditions (i.e. E11.5 XX control, E11.5 XX dko, E11.5 XY control, E11.5 XY dko); the expression value in wild type SF1+ cells between E10.5 and E13.5 and finally gene ontology annotation.
(XLSX)

Table S2 3-ways ANOVA analysis of the sources of gene expression variation for 65 selected genes measured by the Nanostring Ncounter system.
(PDF)

Table S3 Reagents and antibodies.
(PDF)

Table S4 Primer sequences used for Real-Time PCR.
(PDF)
