# Supplementary figures and images for: Insulin and IGF1 Receptors Are Essential for XX and XY Gonadal Differentiation and Adrenal Development in Mice
Source: PLoS Genet. 2013 Jan 3;9(1):e1003160. doi: 10.1371/journal.pgen.1003160 (PMC3536656; doi:10.1371/journal.pgen.1003160)

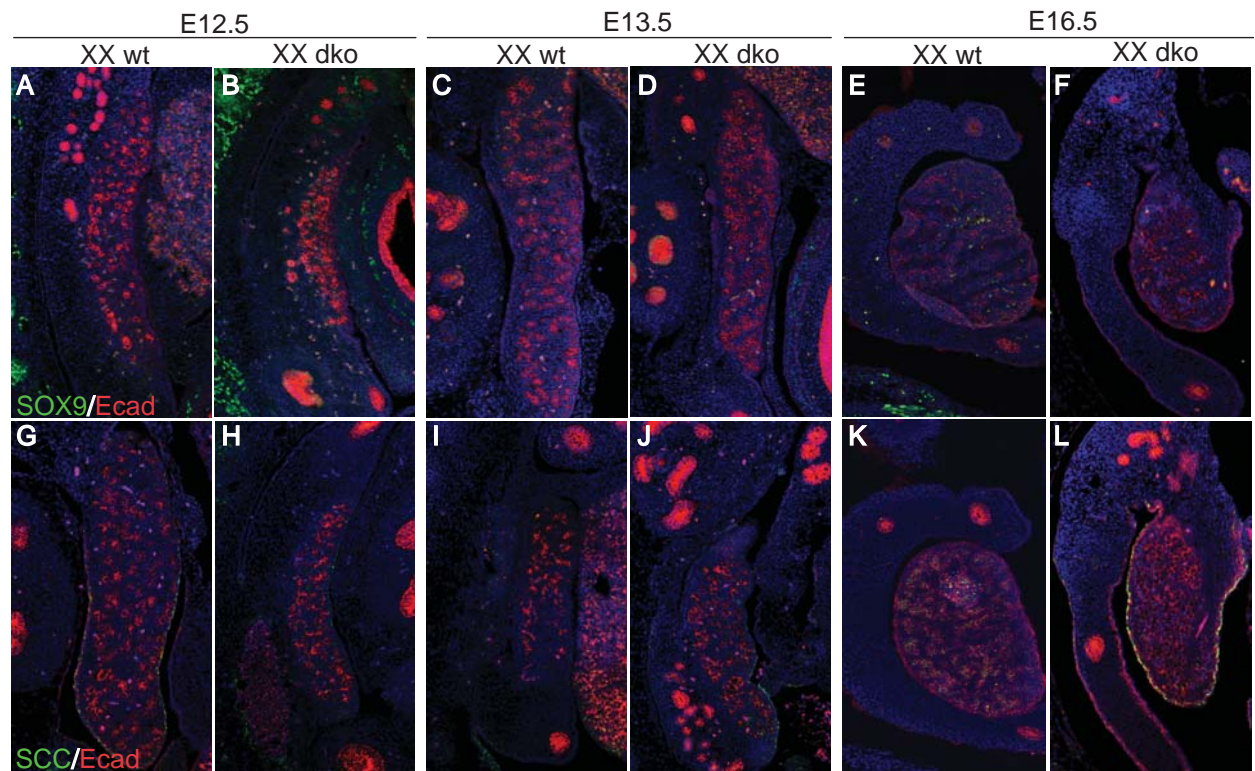

Supplement: Figure S1 — Absence of testicular markers in XX dko gonads. The expression of key testicular markers was assessed in control and dko XX gonads at E12.5, E12.5 and E16.5 with double immunofluorescence using either the Sertoli cell marker SOX9 (A–F, in green) or the Leydig cell marker P450SCC (G–L, in green) along with E–cadherin (red). (PDF) [file pgen.1003160.s001.pdf]

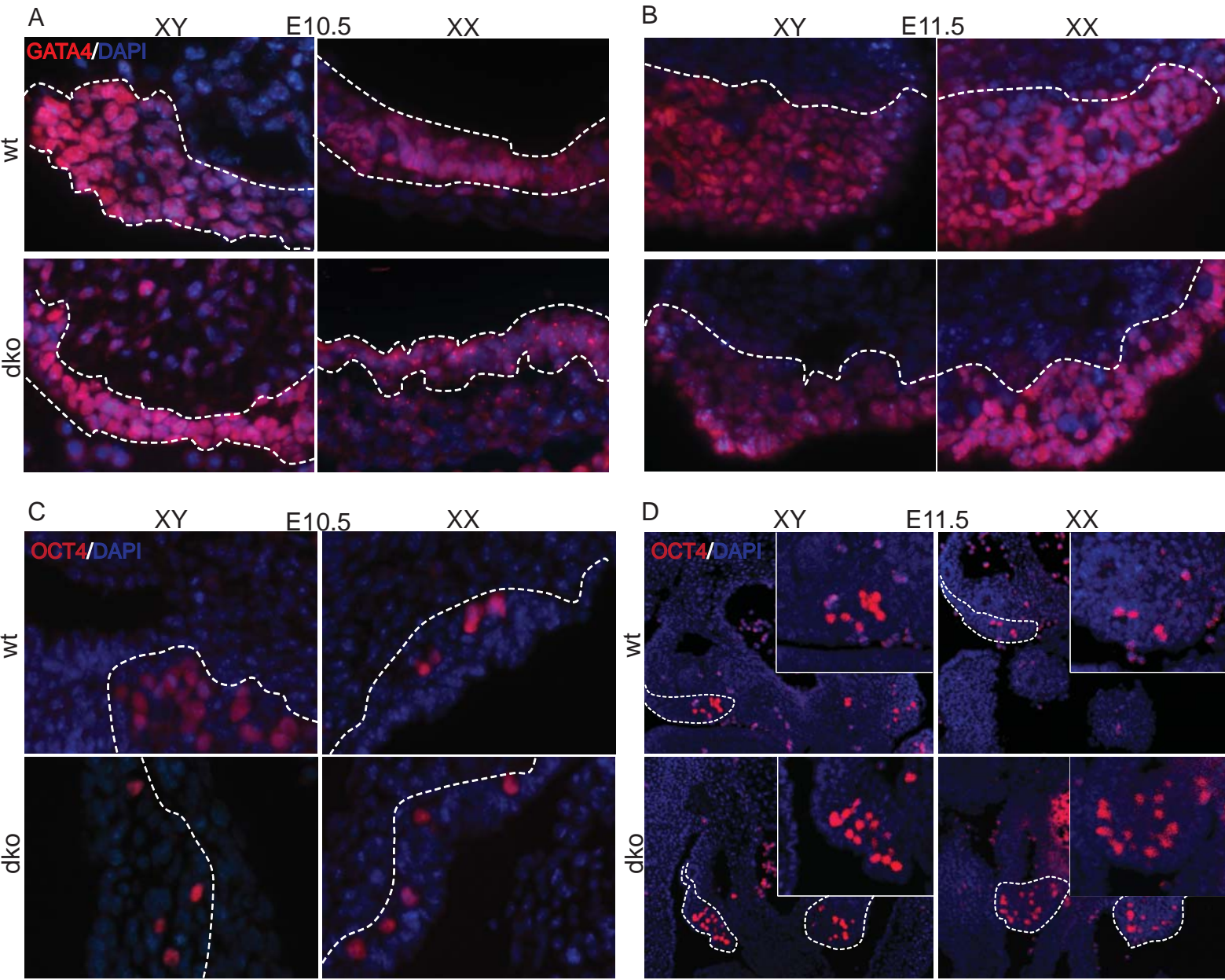

Supplement: Figure S2 — Markers of both somatic progenitors and germ cells are normally present in dko gonads at E10.5 and E11.5. The presence of both somatic precursors and germ cells in control and dko gonads at E10.5 and E11.5 was assessed by immunofluorescence using either the somatic progenitor cell marker GATA4 (A,B, in red) or the germ cell marker OCT4 (C,D, in red). Gonadal tissues are delimited by dotted lines. (PDF) [file pgen.1003160.s002.pdf]

A E11.5

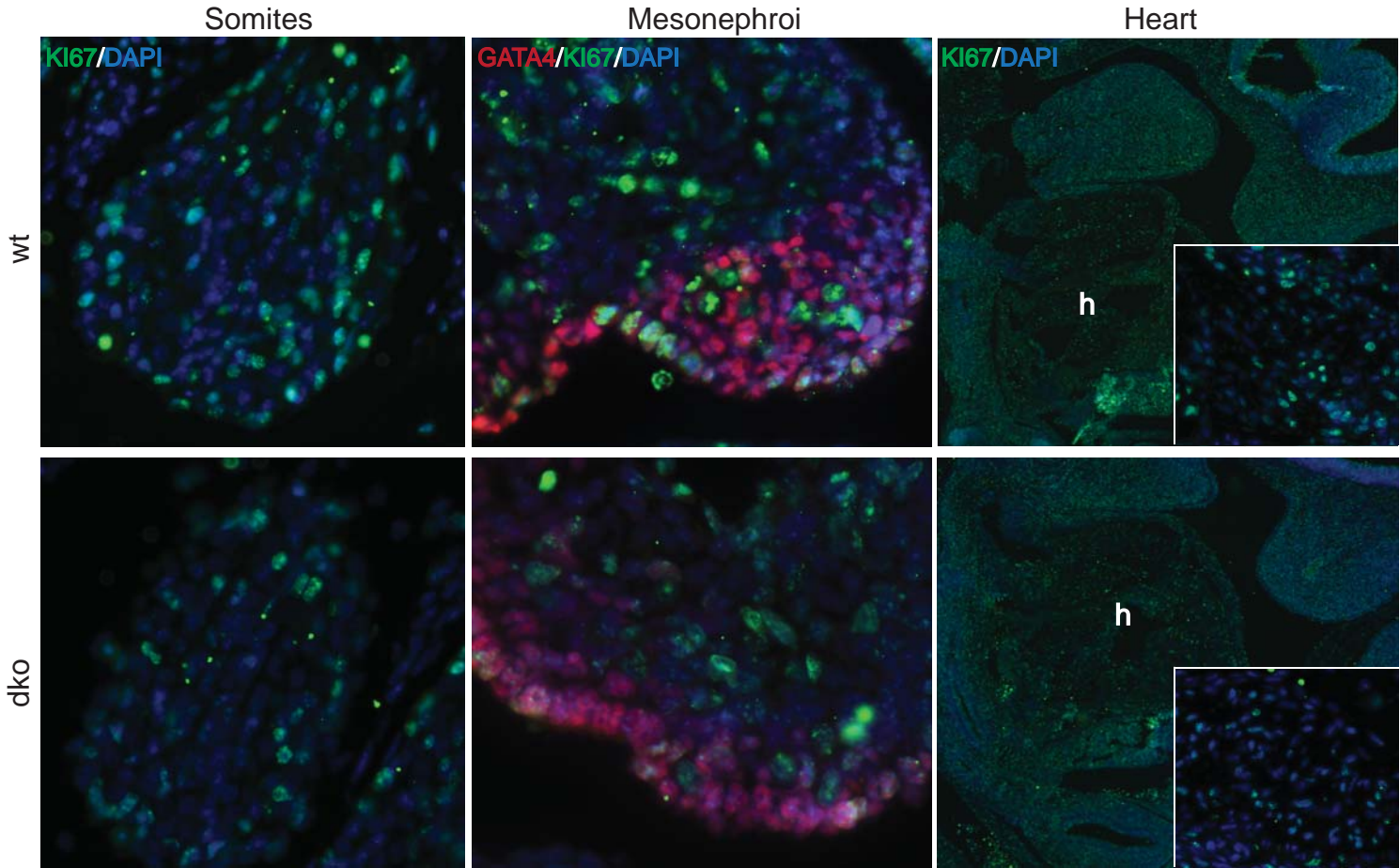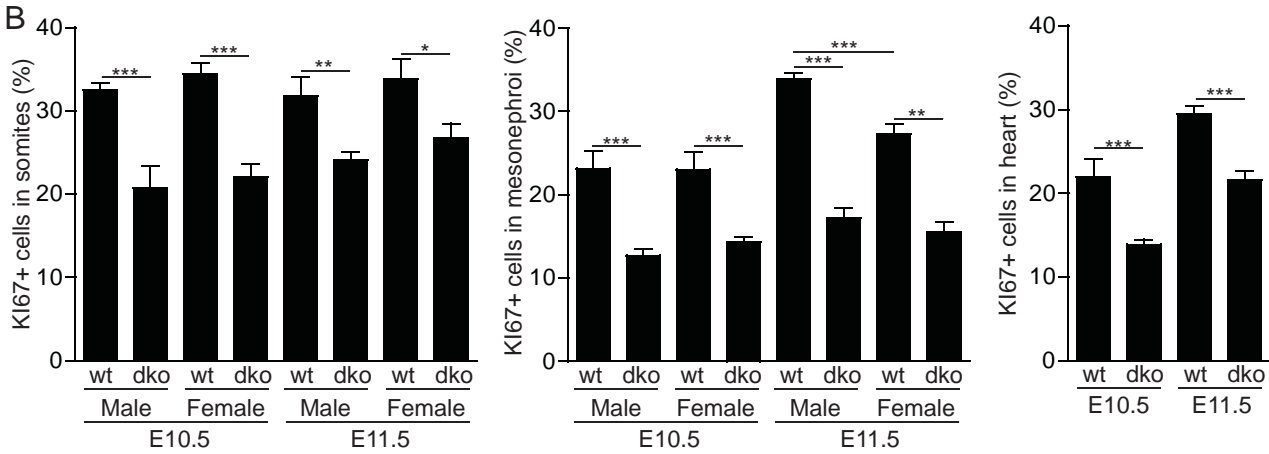

Supplement: Figure S3 — Reduced proliferation rates in somites, mesonephroi and heart of dko embryos at E10.5 and E11.5. (A) Cell proliferation in the developing somites, mesonephroi and heart was evaluated by immunofluorescence using the proliferating marker KI67 (green). In somites and heart, proliferating cells are positive for KI67 (green) and DAPI (blue), whereas in the mesonephros, proliferating cells are KI67+, DAPI+ but GATA4 negative (red). (B) Quantification of KI67+/DAPI+ revealed a significant reduction in the rate of proliferation of the cells composing the somites, the mesonephroi and heart in dko embryos both at E10.5 and E11.5. The proliferation rate was evaluated using three different embryos for each sex, stage and genotype and a minimum of three slides per embryo (n>9). Values are expressed as means ± SEM, p*<0.05, p**<0.01, ***p<0.001 vs control. (PDF) [file pgen.1003160.s003.pdf]

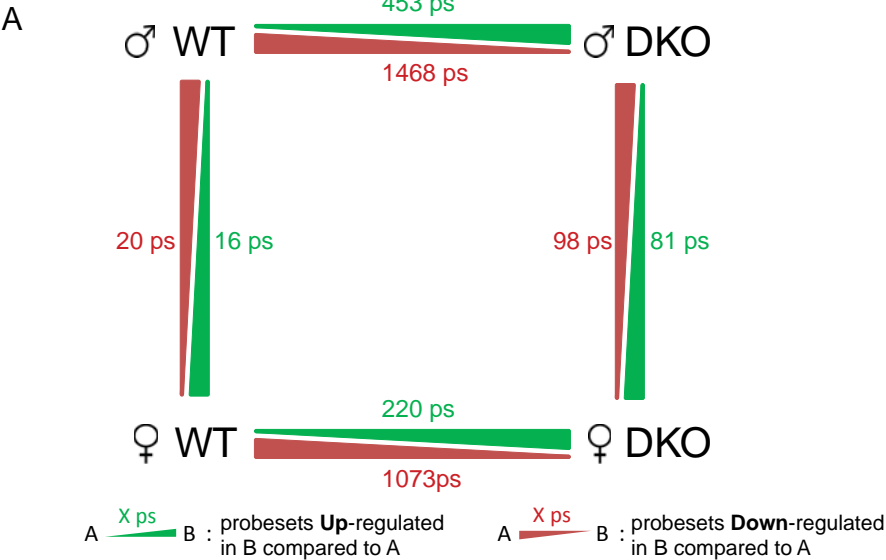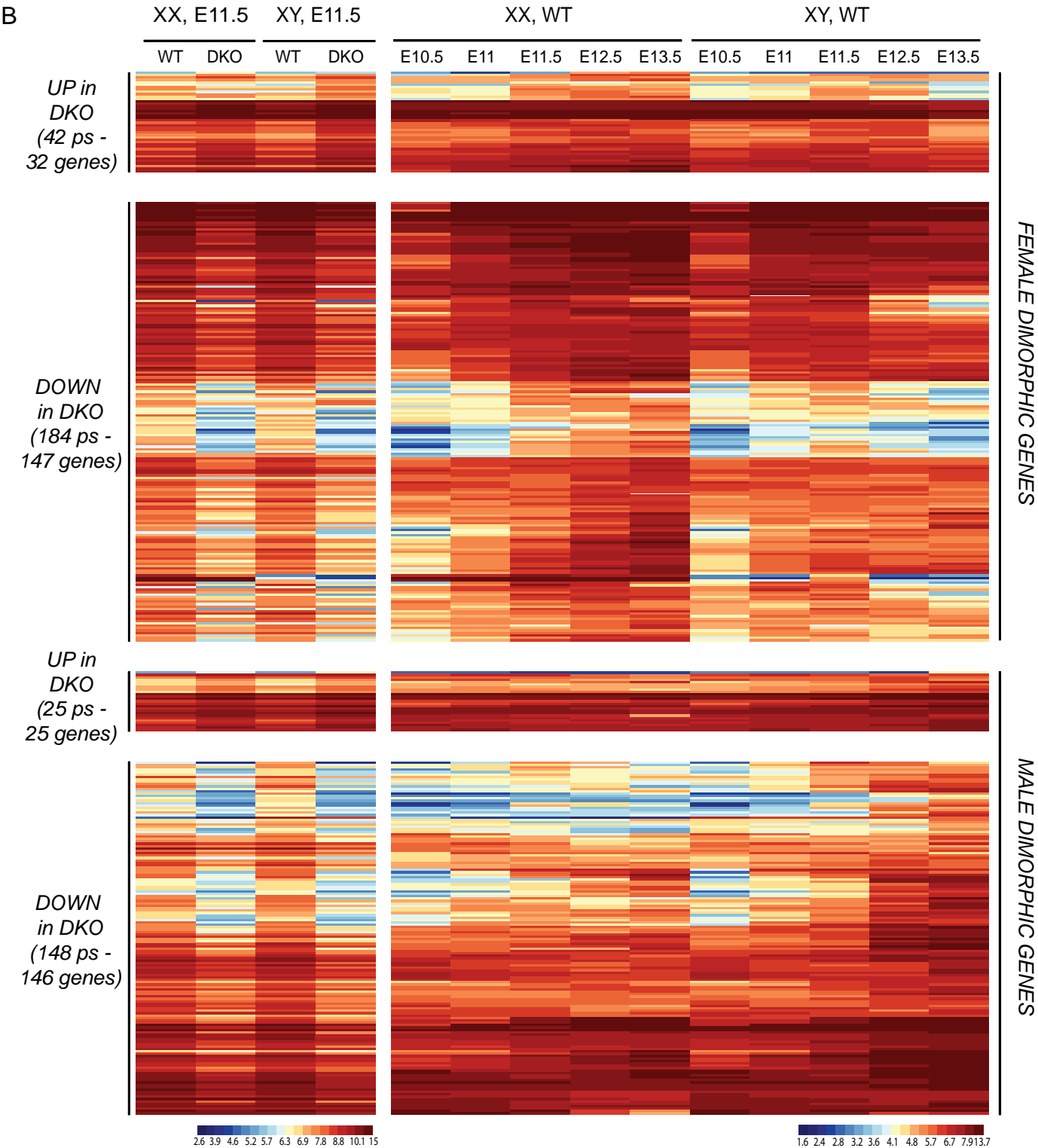

Supplement: Figure S4 — Representation of the 397 probesets being differentially expressed in dko SF1+ cells. (A) Graphic representations of the number of probesets found up- (green) or down-regulated (red) for each of the pairwise comparisons performed between the 4 analyzed samples. (B) Heatmap representation of the probesets exhibiting an altered signal in mutant SF1+ cells and a dimorphic expression pattern between male and female embryonic gonads. Female and male dimorphic genes were clustered into two groups according to the expression profiles described in Nef et al 2005 ([10], indicated on the right). A K-means clustering strategy was performed to identify probesets within these two groups that were up- and down-regulated in dko SF1+ cells (indicated on the left). The first four columns of the heatmap summarize the expression patterns of the 397 probesets within the control (WT) and double mutant (DKO) samples at embryonic day 11.5 (E11.5), whereas the last ten columns show the expression profiles of these probesets in female (XX) and male (XY) between E10.5 and E13.5. Expression levels are indicated by blue (low) or red (high) colouring. (PDF) [file pgen.1003160.s004.pdf]

Pitetti\_Supp\_Fig6

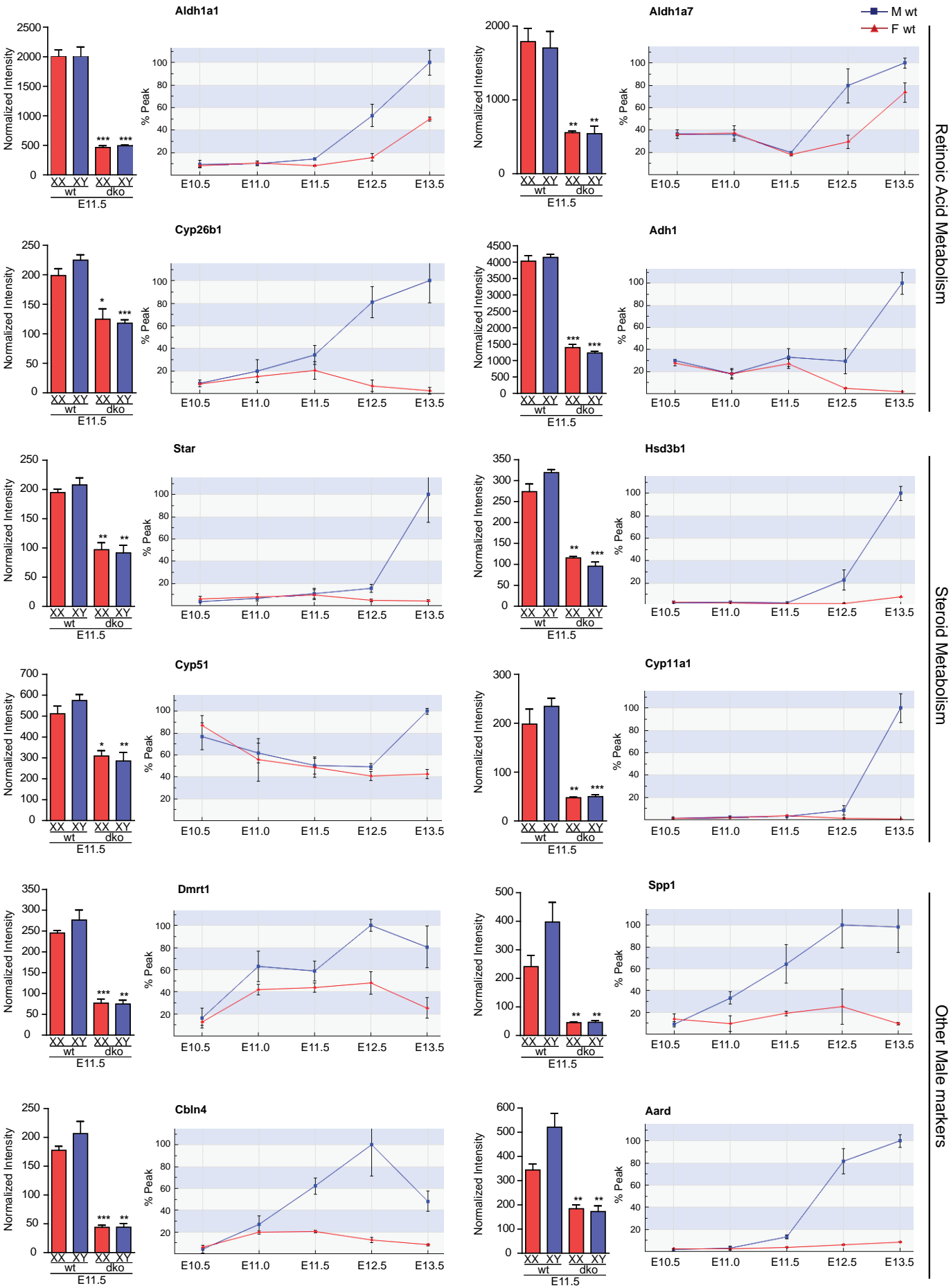

Supplement: Figure S6 — Expression profiles of known male dimorphic genes in dko gonads. Probeset intensities between XX and XY control and dko mutant SF1+ cells at E11.5 were pairwise compared and submitted to an unpaired t-test to evaluate the significance of differences between samples, *p<0.05, **p<0.01, ***p<0.001 vs control. Graphs illustrating the expression profiles of the selected probesets in SF1+ cells at different times of gonad sex determination as determined in Nef et al 2005 ([10]). The peak expression level is set as 100%, and the expression levels at other time points are relative to peak levels (% peak). Bars represent the standard deviation. (PDF) [file pgen.1003160.s006.pdf]

Pitetti\_Supp.Fig7

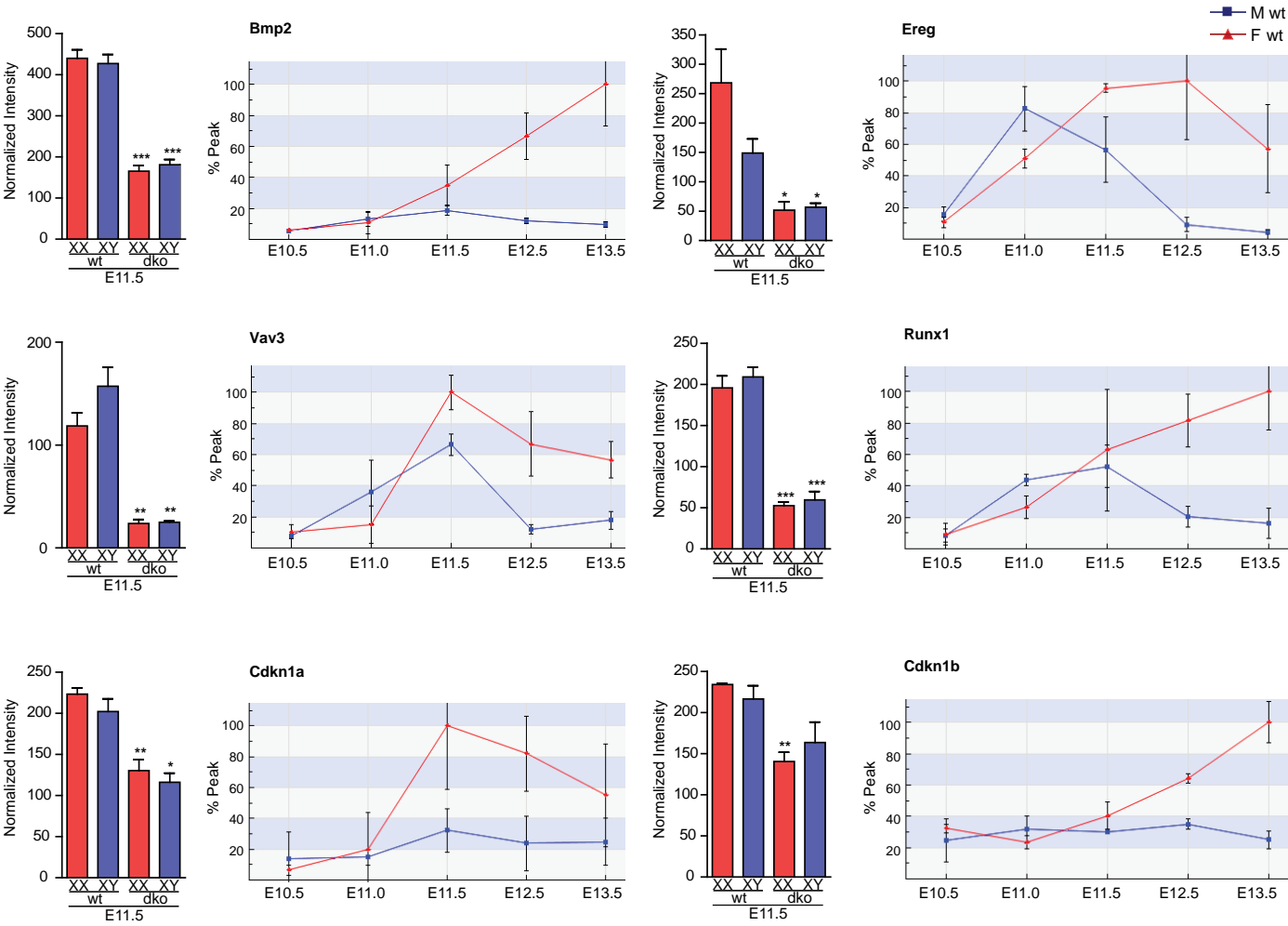

Supplement: Figure S7 — Expression profiles of known female dimorphic genes within the dko gonads. Probeset intensities between XX and XY control and dko mutant SF1+ cells at E11.5 were pairwise compared and submitted to an unpaired t-test to evaluate the relevance of differences between samples, *p<0.05, **p<0.01, ***p<0.001 vs control. Graphs illustrating the expression profiles of the selected probesets in SF1+ cells at different times of gonad sex determination as determined in Nef et al 2005 ([10]). The peak expression level is set as 100%, and the expression levels at other time points are relative to peak levels (% peak). Bars represent the standard deviation. (PDF) [file pgen.1003160.s007.pdf]

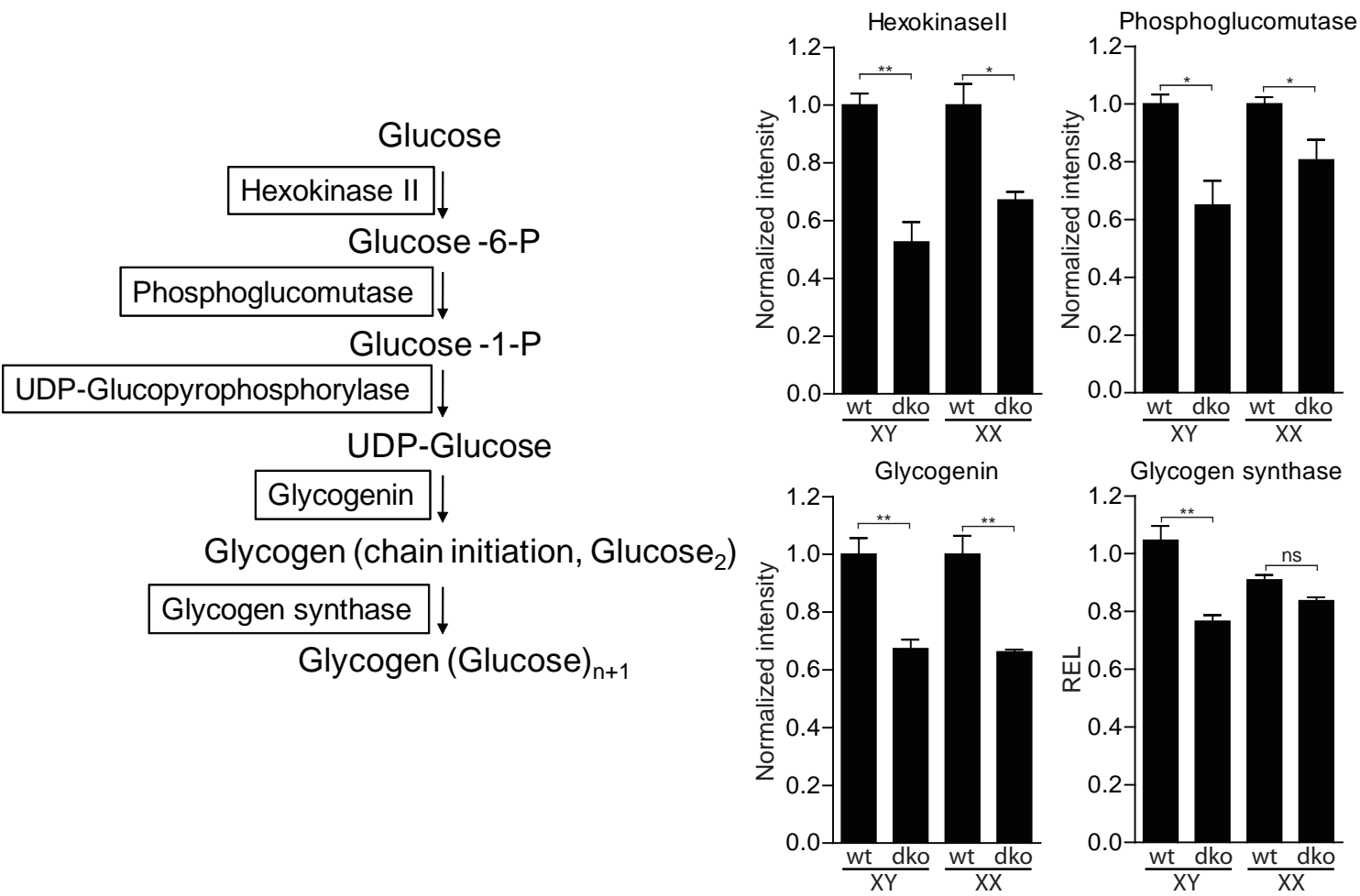

Supplement: Figure S9 — Alteration of genes involved in the glycogen synthesis pathway. Pathway for conversion of glucose monomers to polymeric glycogen including relevant enzymes. Transcript levels for these enzymes were assessed in XY and XX control and dko gonads at E11.5 and E12.5 by Affymetrix analyses or by qRT-PCR. Values are expressed as means ± SEM, **p<0.01, ***p<0.001 vs control. (PDF) [file pgen.1003160.s009.pdf]

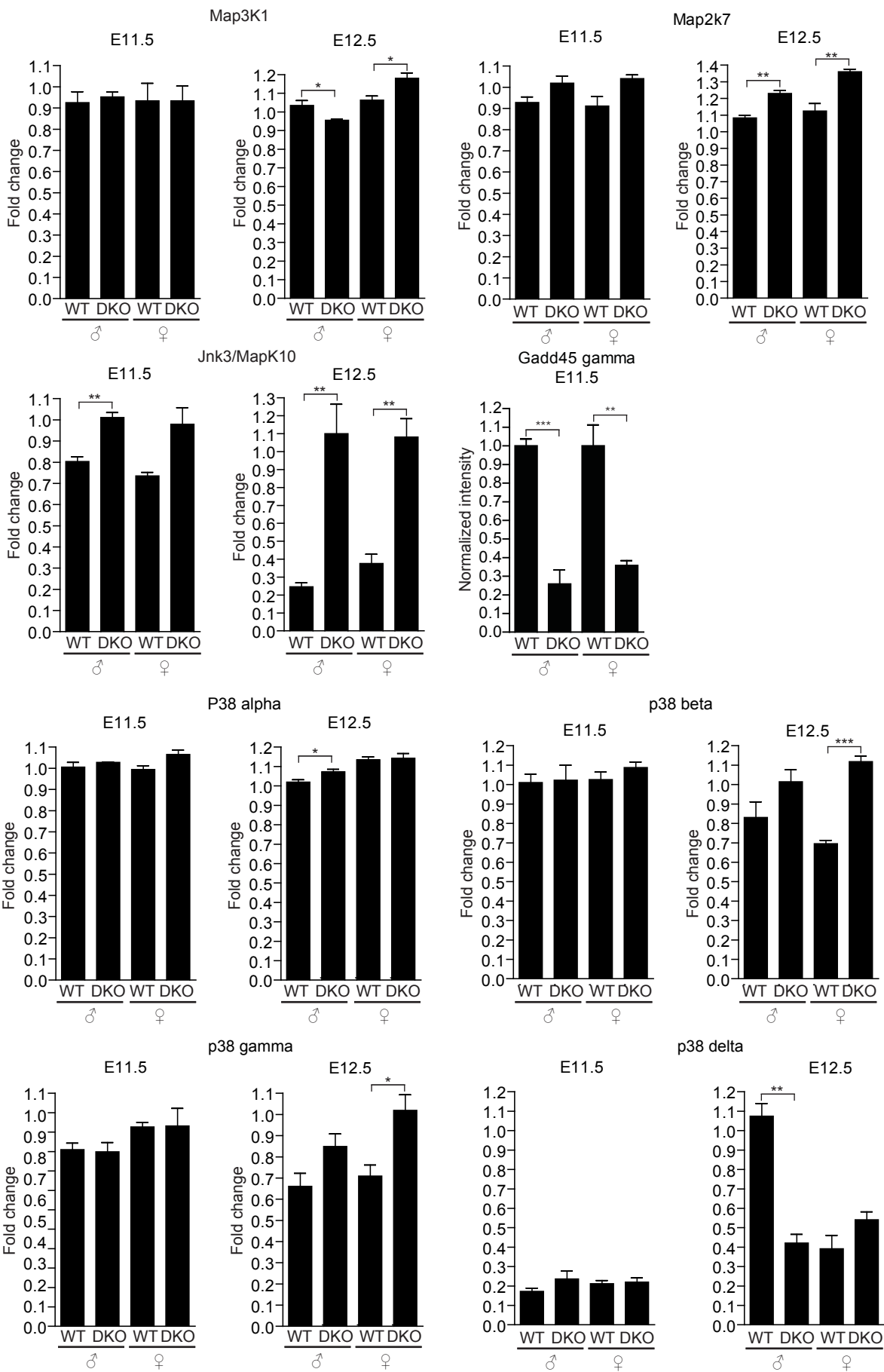

Supplement: Figure S10 — Alteration of genes involved in the MAPK pathway. Transcript levels were assessed in XY and XX control and dko gonads at E11.5 and E12.5 by Affymetrix analyses or by qRT-PCR. Values are expressed as means ± SEM, **p<0.01, ***p<0.001 vs control. (PDF) [file pgen.1003160.s010.pdf]

FACS: scatter plot at E11.5

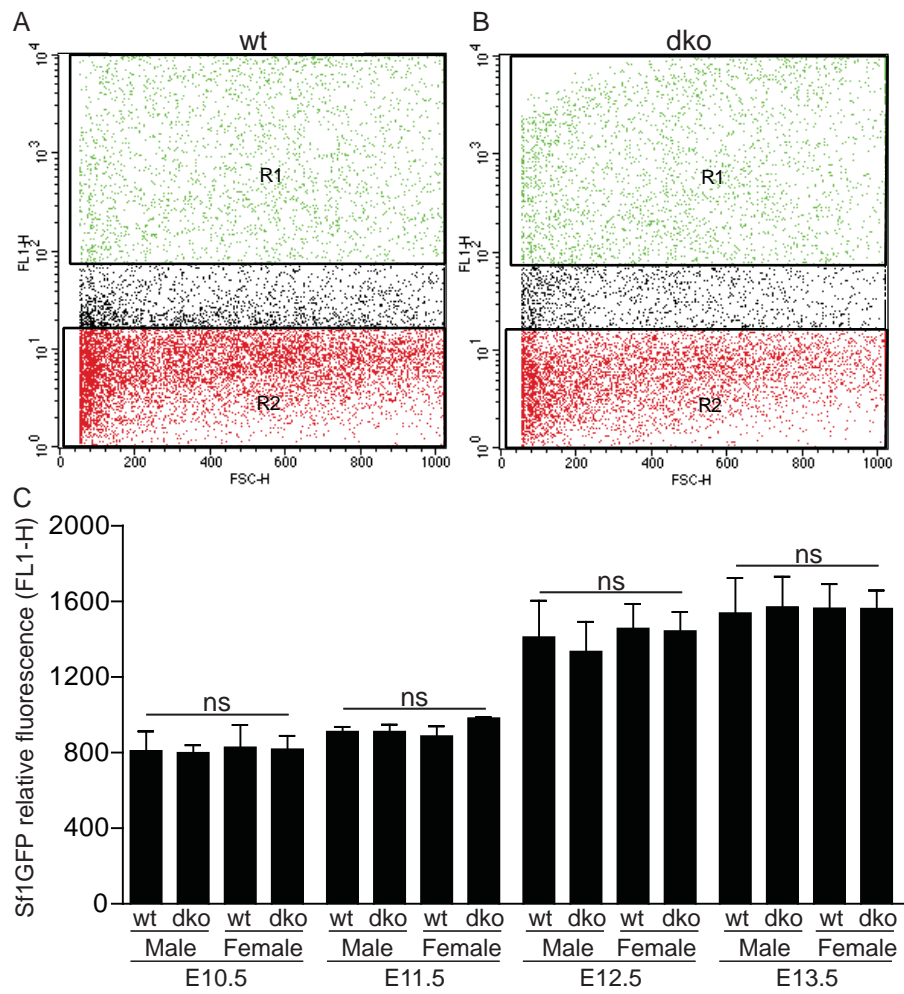

Supplement: Figure S11 — GFP fluorescence of SF1+ cells was not affected by the genetic sex and the genotype of developing embryos. Representative FACS graphs showing the typical and identical pattern of Sf1/eGFP+ cell distribution from control (A) or dko (B) XY genital ridges at E11.5. FL1H axis represents the levels of fluorescence (arbitrary units) while the x axis reflects the size of the cell. (C) Relative levels of Sf1/eGFP fluorescence were not affected by the sex and the genotype. For each condition tested, a mimimum of 6 embryos was analyzed. The nonparametric unpaired t-test was applied for statistical analysis. The abbreviation ns denotes non-significant changes (p>0.05). (PDF) [file pgen.1003160.s011.pdf]

A

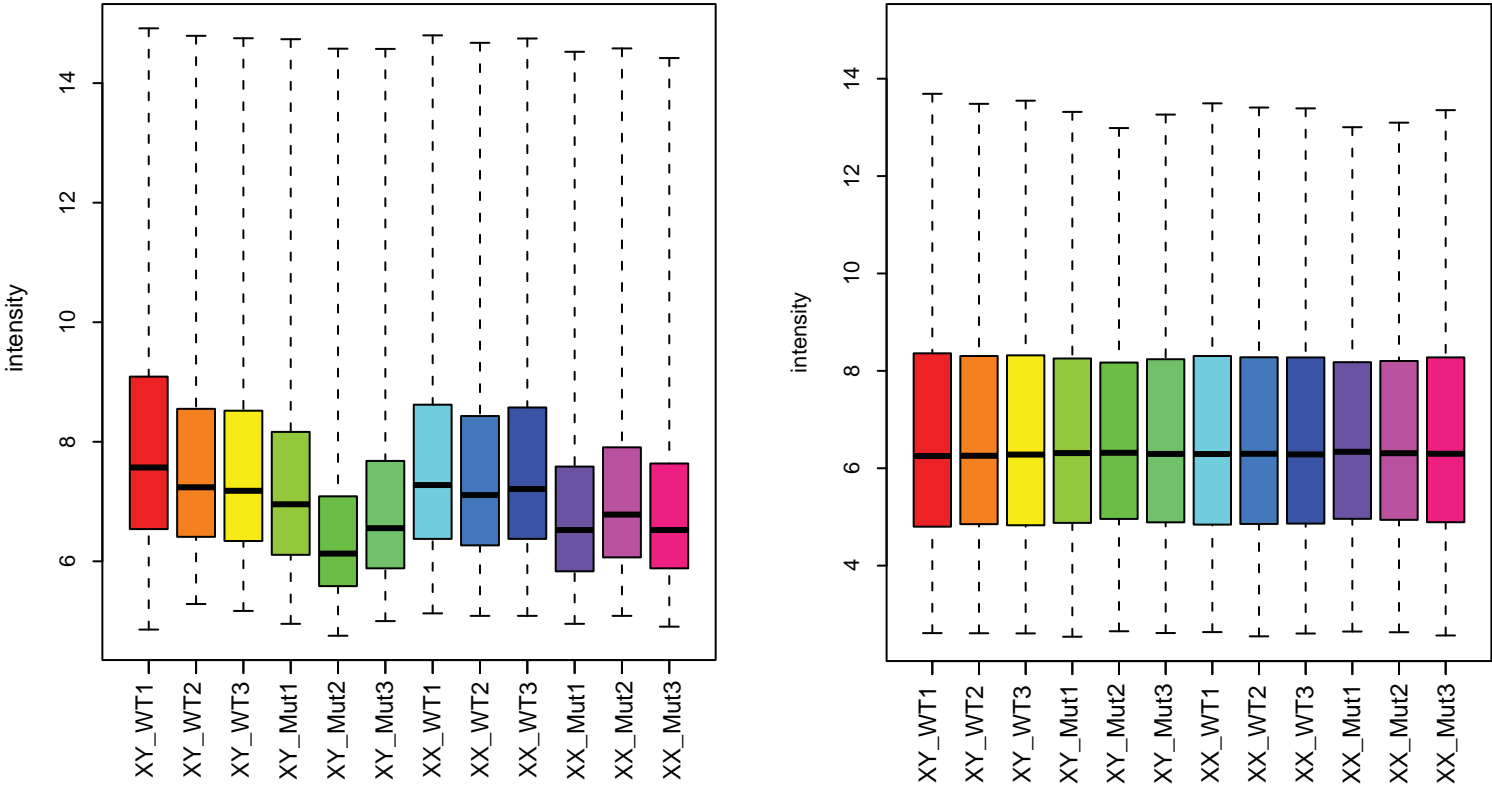

B

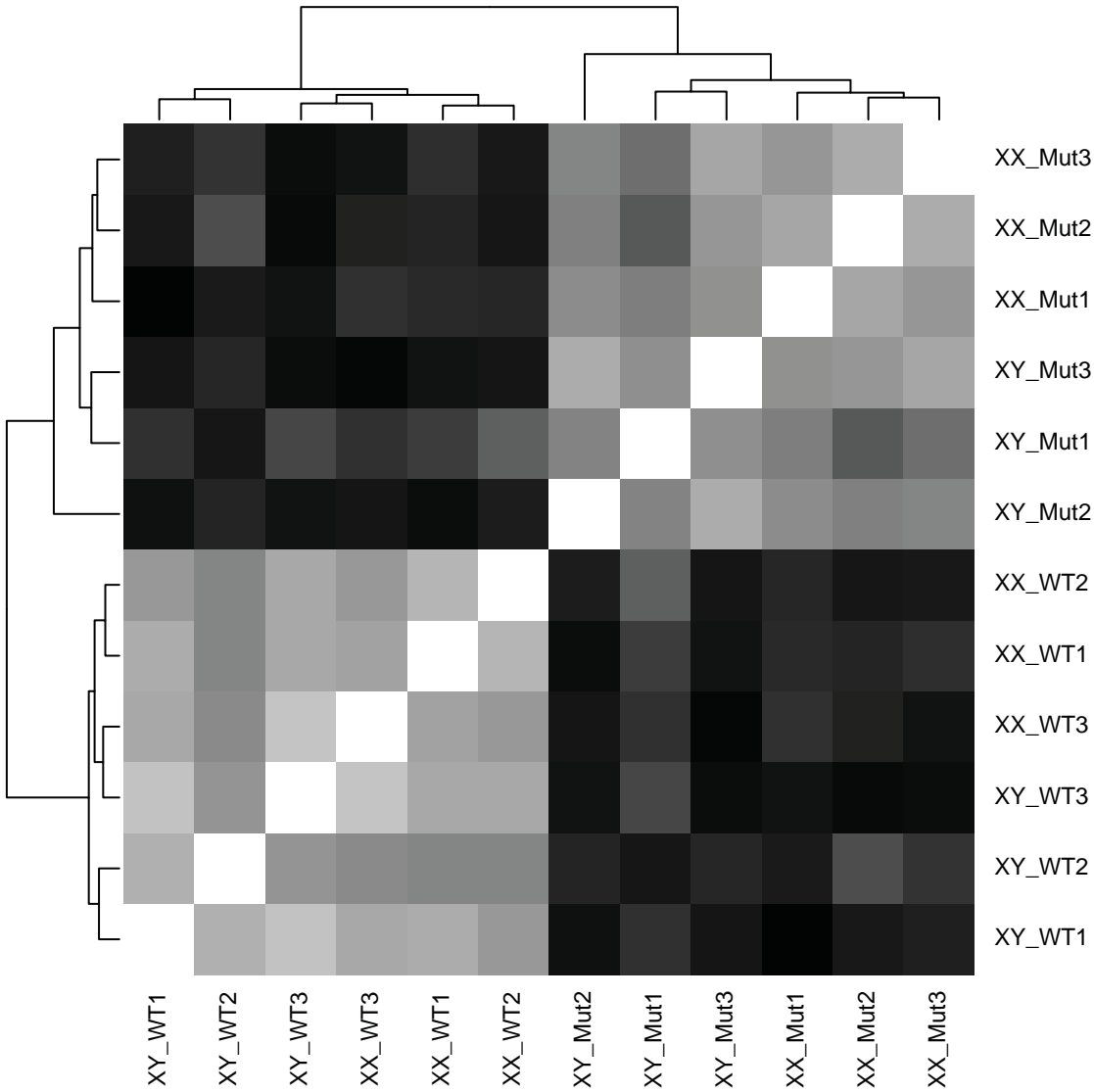

Supplement: Figure S12 — Validation of the RMA normalization process. (A) Box plot representation of expression data before and after RMA normalization. Scale bar indicates Log2-transformed probeset intensities. WT = control; Mut = dko. (B) Correlation matrix for sample duplicates. High and low degrees of similarity between two samples are displayed in black and white, respectively. WT = control; Mut = dko. (PDF) [file pgen.1003160.s012.pdf]
