# Supplementary material for: Insulin and IGF1 Receptors Are Essential for XX and XY Gonadal Differentiation and Adrenal Development in Mice
Source: PLoS Genet. 2013 Jan 3;9(1):e1003160. doi: 10.1371/journal.pgen.1003160 (PMC3536656; doi:10.1371/journal.pgen.1003160)
Supplement: Figure S5 — Genes from known biological processes are affected in dko gonads. Selection of a set of Gene Ontology (GO) terms found enriched within the two groups of up- and down-regulated genes. Each cluster is matched with enriched GO terms from the ontologies “biological process”, “molecular function” and “cellular component”, and the numbers of genes (Entrezgene id) associated with a specific GO term and enriched in each cluster are given within rectangles as observed (left number) and as expected (right number). The color of the rectangle indicates overrepresentation (red) and underrepresentation (blue) as indicated in the scale bar. (PDF) [file pgen.1003160.s005.pdf]

Pitetti_Supp.Fig5

**molecular function**	**18509**	**UP in DKO** **337**	**DOWN in DKO** **1197**
0003700 transcription factor activity	721	**33 / 13**	56 / 47
0043565 sequence-specific DNA binding	615	**29 / 11**	48 / 40
0030528 transcription regulator activity	1244	**48 / 23**	**114 / 81**
0003824 catalytic activity	4766	85 / 87	**414 / 308**
0043560 insulin receptor substrate binding	10	0 / 0	**6 / 1**
0051427 hormone receptor binding	96	0 / 2	**18 / 6**
0043559 insulin binding	6	0 / 0	**4 / 0**

**biological process**	**18675**	**340**	**1206**
0009888 tissue development	825	**36 / 15**	66 / 53
0007399 nervous system development	1097	**55 / 20**	97 / 71
0001501 skeletal system development	330	**21 / 6**	30 / 21
0051216 cartilage development	105	**10 / 2**	6 / 7
0031643 positive regulation of myelination	4	**3 / 0**	0 / 0
0007155 cell adhesion	566	**36 / 10**	42 / 37
0008152 metabolic process	7296	**168 / 133**	**645 / 471**
0033554 cellular response to stress	608	8 / 11	**79 / 39**
0051716 cellular response to stimulus	3431	80 / 63	**286 / 222**
0044267 cellular protein metabolic process	2050	46 / 37	**196 / 132**
0044281 small molecule metabolic process	1756	28 / 32	**153 / 113**
0006629 lipid metabolic process	762	12 / 14	**78 / 49**
0008202 steroid metabolic process	197	1 / 4	**32 / 13**
0006695 cholesterol biosynthetic process	26	0 / 1	**8 / 2**
0006066 alcohol metabolic process	418	2 / 8	**46 / 27**
0000278 mitotic cell cycle	358	3 / 7	**42 / 23**
0022402 cell cycle process	544	8 / 10	**62 / 35**
0006916 anti-apoptosis	156	3 / 3	**27 / 10**
0007548 sex differentiation	182	7 / 3	**25 / 12**
0046545 development of primary female sexual characteristics	87	2 / 2	**17 / 6**
0008406 gonad development	131	5 / 2	**20 / 9**
0048545 response to steroid hormone stimulus	151	4 / 3	**22 / 10**
0043406 positive regulation of MAP kinase activity	106	4 / 2	**18 / 7**
0045860 positive regulation of protein kinase activity	247	10 / 5	**31 / 16**

**cellular component**	**18880**	**345**	**1219**
0031012 extracellular matrix	320	**18 / 6**	22 / 21
0005925 focal adhesion	99	**9 / 2**	12 / 6
0005634 nucleus	4509	**138 / 82**	**447 / 291**
0044444 cytoplasmic part	5163	107 / 94	**448 / 333**
0005739 mitochondrion	1365	18 / 25	**118 / 88**

Depleted **p-value** **Enriched**

1 **0.95** **0.05** **1e-5** **0**
