# Supplementary material for: Insulin and IGF1 Receptors Are Essential for XX and XY Gonadal Differentiation and Adrenal Development in Mice
Source: PLoS Genet. 2013 Jan 3;9(1):e1003160. doi: 10.1371/journal.pgen.1003160 (PMC3536656; doi:10.1371/journal.pgen.1003160)

Pitetti_Supp.Fig8A

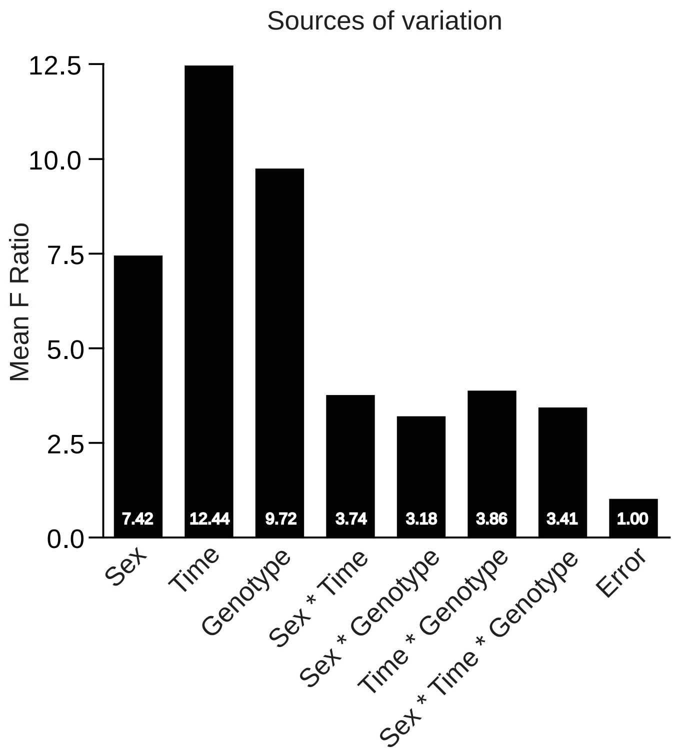

Pitetti_Supp.Fig8B (I) Male genes

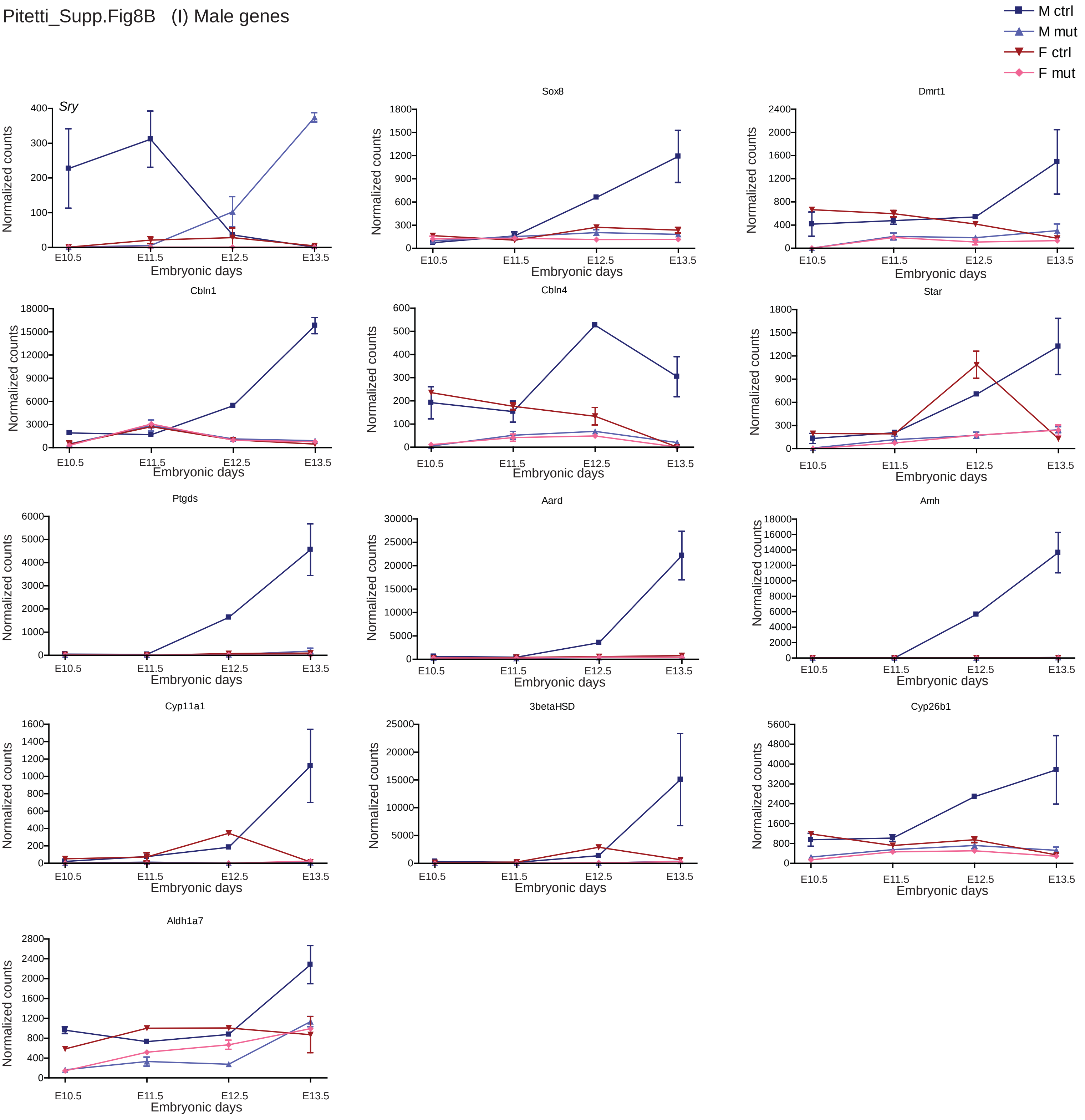

Pitetti_Supp.Fig8B (II) Female genes

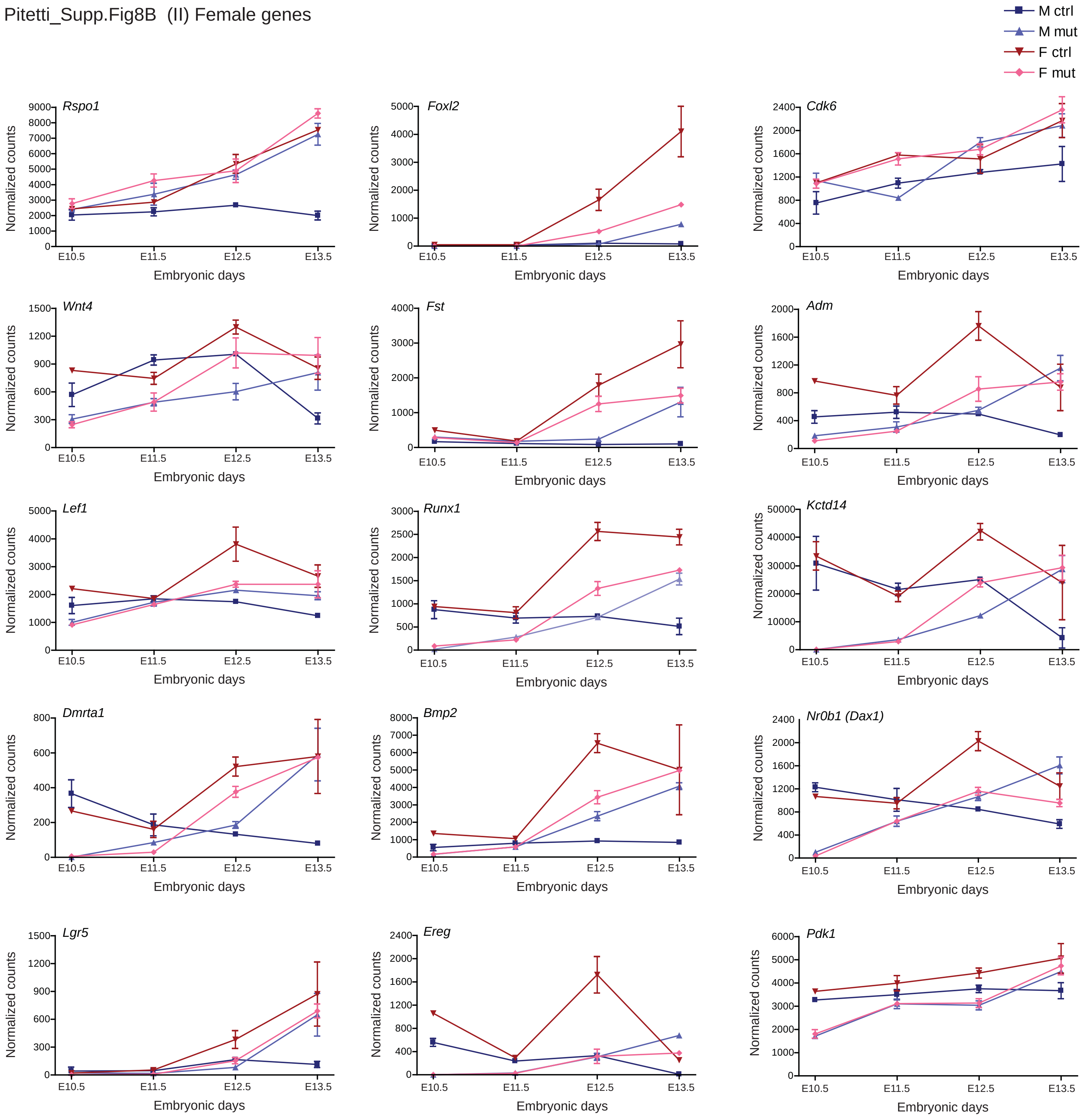

Pitetti_Supp.Fig8B (III) Adreno-gonadal development genes

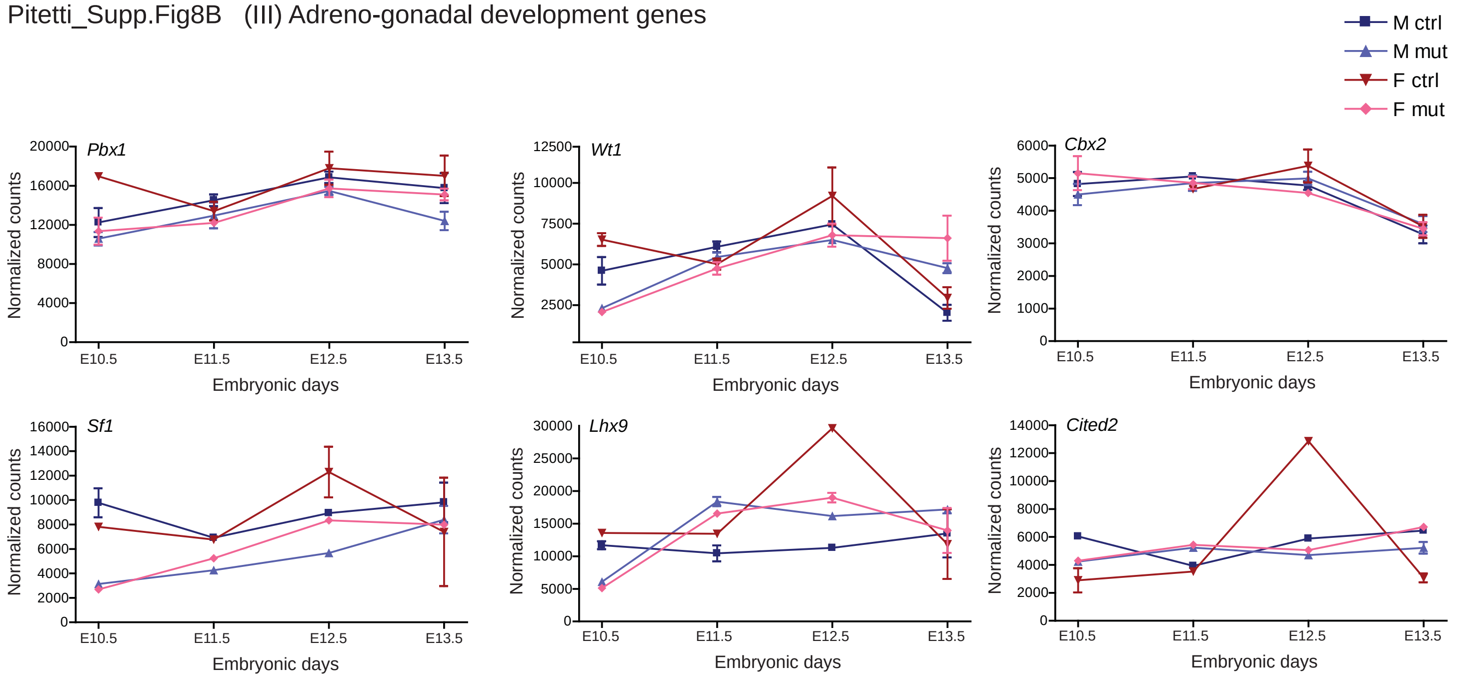

Pitetti_Supp.Fig8B (IV) Miscellaneous genes

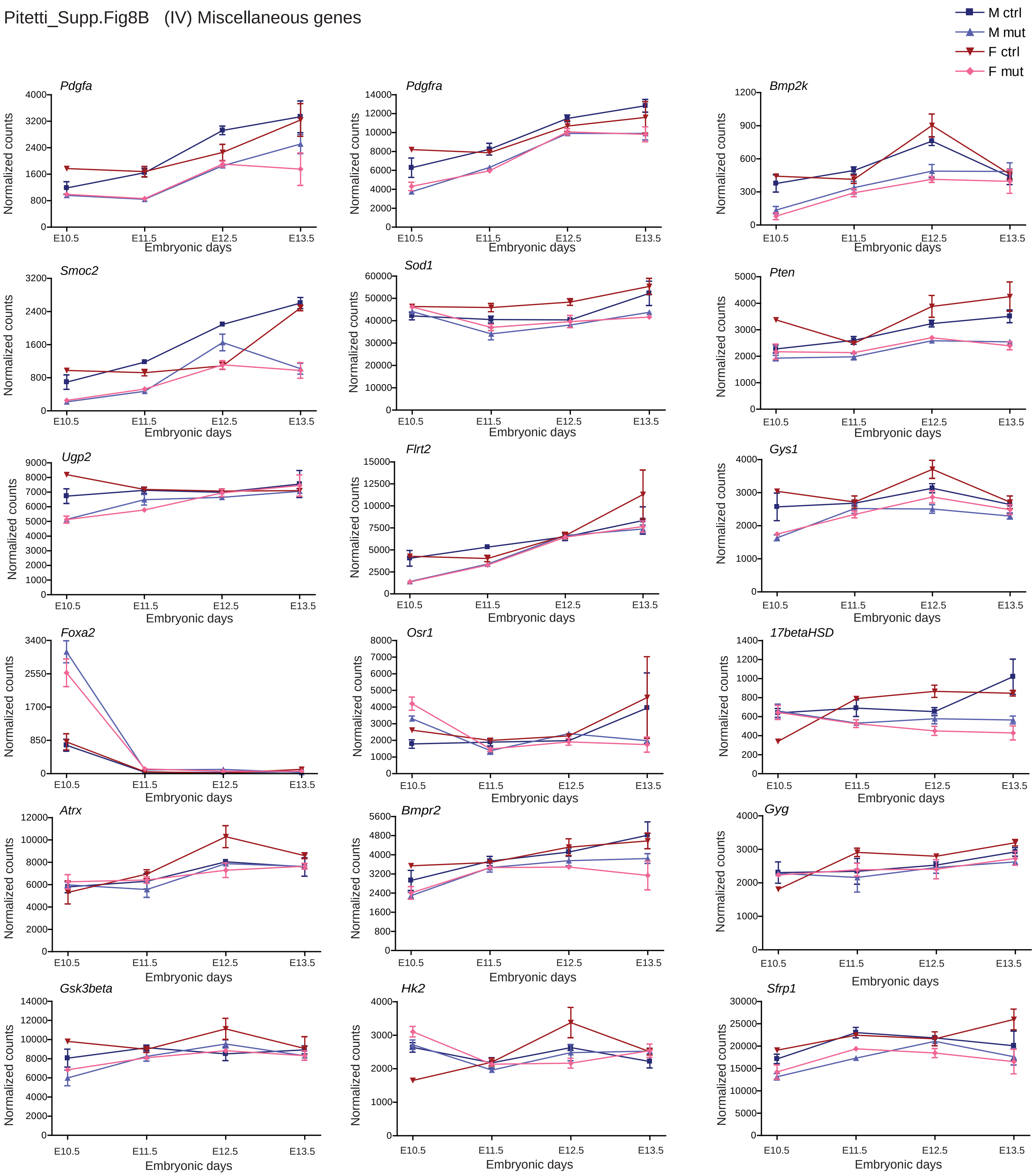

Pitetti_Supp.Fig8B (V) Insulin/Igfs signaling pathway associated genes

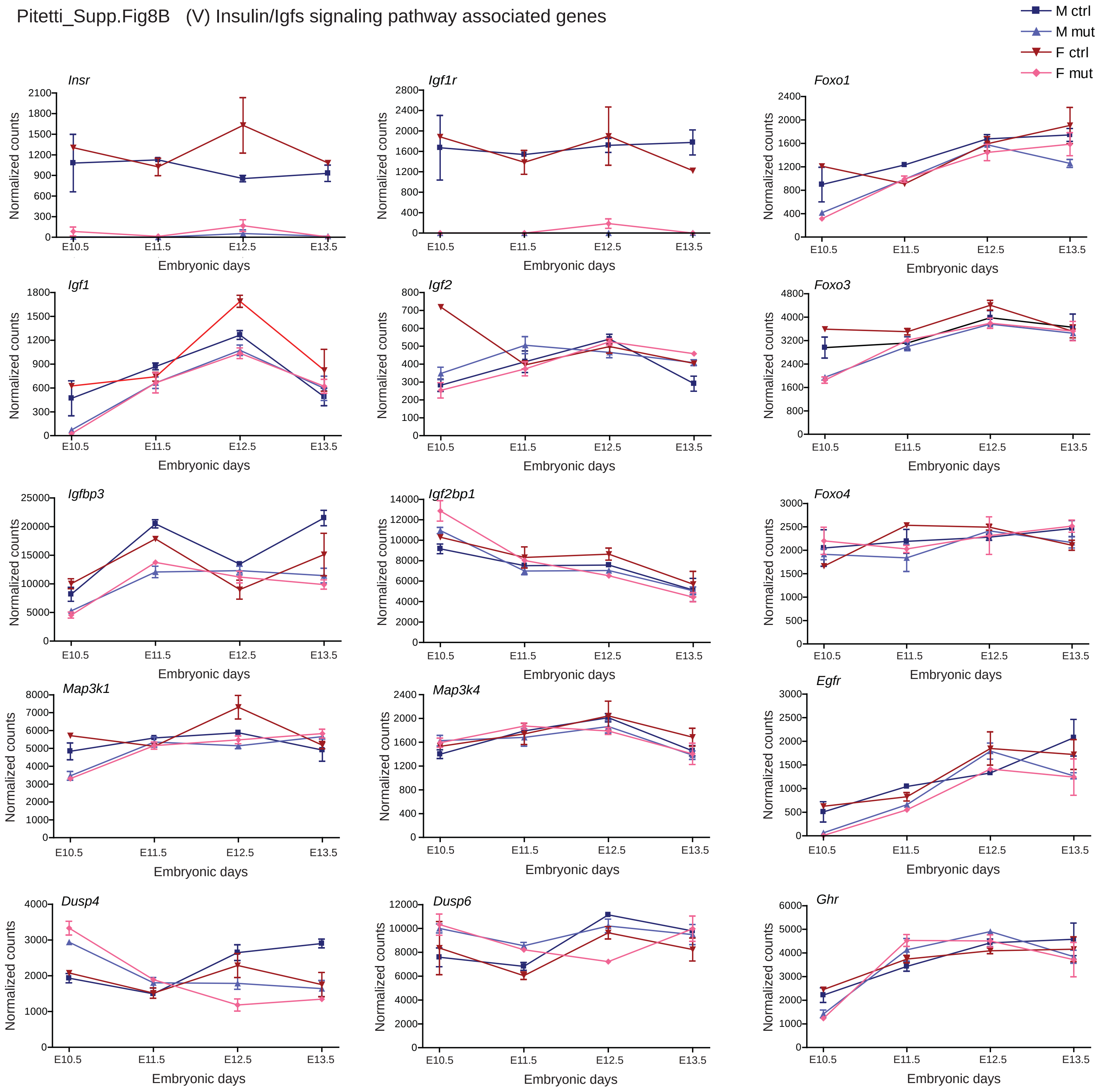

Supplement: Figure S8 — Expression profiles of 65 genes as determined by NanoString Multiplex Assays. (A) Analysis of the sources of variation (ANOVA) revealed that the main sources of variations are genotype, sex and time. (B) The set of selected genes includes classical genes involved in the testicular program, the ovarian program, adrenogonadal development and insulin/IGF signaling as well as a selection of CAGP and primed genes. Total RNAs were isolated from SF1+ cells isolated from XX and XY control or dko gonads between E10.5 and E13.5 control female mice (red); control male mice (blue); dko female mice (light red); dko male mice (light blue). Bars represent the standard deviation. (PDF) [file pgen.1003160.s008.pdf]
