# Supplementary material for: Insulin and IGF1 Receptors Are Essential for XX and XY Gonadal Differentiation and Adrenal Development in Mice
Source: PLoS Genet. 2013 Jan 3;9(1):e1003160. doi: 10.1371/journal.pgen.1003160 (PMC3536656; doi:10.1371/journal.pgen.1003160)
Supplement: Table S2 — 3-ways ANOVA analysis of the sources of gene expression variation for 65 selected genes measured by the Nanostring Ncounter system. (PDF) [file pgen.1003160.s014.pdf]

Pitetti_Supp.Table2

Statistical analysis of the sources of gene expression varation for 65 selected genes measured by Nanostring Ncounter system

Type	Gene name	p-value(Sex)	p-value(Time)	p-value(Genotype)	p-value(Sex * Time)	p-value(Sex * Time * Genotype)	p-value(Sex * Genotype)	p-value(Time * Genotype)
Male	Aard	7.89E-07	7.22E-06	0.00887257	0.004268	0.0205069	0.0217902	0.00011058
	Aldh1a7	0.00056868	4.31E-06	0.0309891	2.15E-05	0.224282	0.220524	0.550698
	Amh	0.000518285	7.31E-08	0.00289804	0.0107594	0.0646822	0.180553	0.00923114
	Cbln1	4.50E-06	0.000372044	0.196203	0.00298621	0.0545865	0.019468	0.0501541
	Cbln4	0.00274205	8.14E-05	0.0016722	0.242616	0.208109	0.0996358	0.515807
	Cyp11a1	0.264428	0.0155193	7.32E-10	0.845861	0.426172	0.928067	0.224844
	Cyp26b1	2.98E-06	5.55E-07	0.0421888	0.00468659	0.080635	0.04974	0.0671024
	Dmrt1	0.0580965	9.49E-13	2.91E-06	0.410085	0.275815	0.899191	0.063408
	Hsd3b1	0.0973257	0.000158052	7.23E-06	0.220929	0.435141	0.673718	0.500304
	Ptgds	2.03E-05	0.00192321	3.48E-06	0.0891011	0.171488	0.188442	0.00936792
	Sox8	0.00027714	4.26E-06	0.446624	0.144152	0.219304	0.0819604	0.000301759
	Sry	1.23E-12	0.00176406	0.00719349	0.162085	2.26E-07	0.954546	5.06E-07
	Star	0.13588	1.33E-08	0.00119045	0.0765408	0.382316	0.814689	0.0350754
Female	Adm	0.0887851	0.000452108	0.900199	0.00633271	0.0034318	0.445011	0.471592
	Bmp2	0.441678	1.32E-12	0.117919	0.000912473	0.000715452	0.646563	0.208184
	Cdk6	0.0164219	2.65E-07	4.86E-05	0.000769984	0.000997711	0.0176162	0.000456346
	Dmrta1	0.522985	9.02E-13	0.0224719	0.0436692	0.00987406	0.556226	8.53E-05
	Ereg	0.282433	0.00911543	0.340135	0.505364	0.105101	0.218234	0.0282869
	Foxl2	0.000230462	4.51E-18	0.23971	0.00484295	0.0220442	0.00129153	7.04E-05
	Fst	0.0200159	4.07E-06	8.23E-06	1.96E-06	0.000341439	0.204396	0.00225453
	Kctd14	0.431103	5.28E-10	0.000219814	0.253829	0.972863	0.782705	0.000179869
	Lef1	0.0260741	9.24E-07	0.0537444	0.000867035	0.000776362	0.105904	0.00637517
	Lgr5	0.330619	4.47E-12	0.354437	0.00892831	0.920396	0.670121	0.26556
	Nr0b1	0.00285412	1.51E-06	0.166295	0.00255933	0.0415531	0.508838	0.837744
	Pdk1	0.0191171	0.107605	1.73E-05	0.0643183	0.0840302	0.041303	0.000164227
	Rspo1	0.510969	0.0111562	0.00221638	0.108557	0.0803197	0.35581	0.130151
	Runx1	0.542254	1.90E-13	0.240942	0.381735	5.56E-05	0.0760154	0.298945
	Wnt4	0.0521346	7.42E-07	0.0851581	0.000190486	0.00179602	0.0328074	0.0256254
AGP	Cbx2	0.0477313	0.0218862	0.0363446	0.0274435	0.0124478	0.0450702	0.0211031
	Cited2	0.126082	0.066628	0.0774061	0.0403257	0.0432272	0.0855457	0.097535
	Lhx9	0.271766	0.0618636	0.142397	0.0940883	0.169923	0.360752	0.431799
	Pbx1	0.190979	0.0158617	0.258422	0.142976	0.1391	0.11847	0.159921
	Sf1	0.201178	0.121648	0.730323	0.135643	0.217516	0.188013	0.125467
	Wt1	0.426215	0.0151828	0.162897	0.241373	0.248678	0.295092	0.485728
Miscellaneous	Atrx	0.10927	0.0255085	0.286786	0.137331	0.0917654	0.216659	0.0713447
	Bmp2k	0.00139918	1.58E-10	0.871399	0.000290676	0.0122113	0.0616359	0.378319
	Bmpr2	0.00327042	3.89E-06	0.0314133	0.000639573	0.000418747	0.00544623	0.000738944
	Flrt2	0.323805	9.23E-07	0.861583	0.176892	0.221571	0.303094	0.771204
	Foxa2	0.484583	8.84E-08	0.117504	0.229149	0.664491	0.822675	0.0558992
	Gsk3b	0.150403	0.0330295	0.214115	0.109559	0.0788842	0.127951	0.112725
	Gyg	0.002599	0.00446397	0.412631	0.00921894	0.0018164	0.272565	0.00118454
	Gys1	0.156417	0.00535276	0.526522	0.0824964	0.0807416	0.0847577	0.199916
	Hk2	0.00273634	0.000438468	0.00231123	7.05E-05	2.92E-05	0.00166137	1.28E-05
	Osr1	0.0192175	0.0164746	0.00962196	0.00267254	0.000649032	0.0153359	7.24E-06
	Pdgfa	0.0288712	1.42E-06	0.922405	0.0888463	0.0465993	0.0965798	0.0157954
	Pdgfra	0.0869173	0.000423024	0.723246	0.211501	0.154334	0.0581883	0.256742
	Pten	0.0123291	1.24E-05	0.0835117	0.000874204	0.000565263	0.00832098	0.000387927
	Sfrp1	0.132176	0.0154975	0.369763	0.113481	0.0842271	0.136312	0.12951
	Sod1	0.153024	0.200898	0.34625	0.169299	0.141882	0.207599	0.0859032
	Ugp2	0.141406	0.0606518	0.361768	0.170757	0.1641	0.147049	0.255649

Pitetti_Supp.Table2

Statistical analysis of the sources of gene expression varation for 65 selected genes measured by Nanostring Ncounter system

Type	Gene name	p-value(Sex)	p-value(Time)	p-value(Genotype)	p-value(Sex * Time)	p-value(Sex * Time * Genotype)	p-value(Sex * Genotype)	p-value(Time * Genotype)
Ins.Sig.Path	Dusp4	0.000715936	0.00370326	0.00528656	0.00131239	0.000420082	0.00154399	5.34E-06
	Dusp6	0.0768455	0.182794	0.0454587	0.218951	0.171021	0.0998682	0.0710637
	Egfr	2.03E-05	1.84E-18	0.0113278	6.45E-07	0.85583	0.710896	0.0712632
	Foxo1	0.00810613	1.69E-10	0.154744	0.00118197	0.00676364	0.0129484	0.0465197
	Foxo3	0.0988645	0.00166643	0.331153	0.0648739	0.0833001	0.103211	0.263256
	Foxo4	0.00251707	0.0063528	0.348191	0.0143606	0.00257266	0.186884	0.00173544
	Ghr	0.0487523	3.40E-06	0.280275	0.0614548	0.106606	0.0728232	0.358894
	Igf1	0.00152079	4.68E-11	0.0600944	0.000877478	0.32217	0.432707	0.29503
	Igf1r	0.350868	0.00475822	2.02E-20	0.0951514	0.0543006	0.500558	0.278615
	Igf2	0.0159399	4.03E-06	0.00664728	0.00284878	0.0112539	0.111157	0.0116656
	Igf2bp1	0.124855	0.111828	0.0904877	0.0937187	0.0565931	0.096018	0.0225897
	Igfbp3	0.150048	0.000196632	0.817481	0.25278	0.263471	0.24321	0.268051
	Insr	0.940791	0.0104261	3.61E-18	0.666519	0.0903757	0.140456	0.0391319
	Map3k1	0.00547486	1.25E-05	0.0144828	0.000275904	0.000368284	0.00558629	0.0015546
	Map3k4	0.00187437	5.88E-05	0.0390162	0.00126243	0.00040437	0.0399648	0.000129152

Statistical analysis:

Concerning the NanoString Ncounter gene expression analysis, we built an Anova model in Partek Genomic suite (Partek inc, St Louis (MI) USA) to estimate the importance of each parameter. In this model we included the time, sex, genotype, and all combined interactions. The equation of the linear model is: Yijklmn = μ + Sexi+Timej+Genotypek+Tissuesl+Replicatem+Sex * Timeij+Sex * Time * Genotype * Tissuesijkl+Sex * Genotypeik+Sex * Tissuesil+Time * Genotypejk+Time * Tissuesjl+Genotype * Tissueskl+ εijklmn. Where Yijklmn represents the nth observation on the ith Sex jth Time kth Genotype lth Tissues mth Replicate. μ is the common effect for the whole experiment. εijklmn represents the random error present in the nth observation on the ith Sex jth Time kth Genotype lth Tissues mth Replicate. The errors εijklmn are assumed to be normally and independently distributed with mean 0 and standard deviation δ for all measurements. Replicate is a random effect. Then we calculate the effect of each factor as described in (Eisenhart 1947).
