# Supplementary material for: Insulin and IGF1 Receptors Are Essential for XX and XY Gonadal Differentiation and Adrenal Development in Mice
Source: PLoS Genet. 2013 Jan 3;9(1):e1003160. doi: 10.1371/journal.pgen.1003160 (PMC3536656; doi:10.1371/journal.pgen.1003160)
Supplement: Table S3 — Reagents and antibodies. (PDF) [file pgen.1003160.s015.pdf]

SUPPLEMENTARY TABLE 3: Reagents and antibodies

Primary antibodies:

Protein targeted	Host	Dilution	Clone	Provider	Catalogue number
SRY	Rabbit	-	-	Dagmar Wilhelm (Aus)	
SOX9	Rabbit	-	-	Dagmar Wilhelm (Aus)	
3β-HSD	Rabbit	1:1000	-	Kenichirou Morohashi (Kyushu, Japan)	
P450SCC	Rabbit	1:200	-	Dagmar Wilhelm (Aus)	
FOXL2	Rabbit	1:1000	-	Dagmar Wilhelm (Aus)	
WT1	Mouse	1:500	-	DakoCytomation (CA, USA)	
SCP3	Mouse	1:200	-	Abcam (USA)	Ab15092
OCT4	Mouse	1:200	-	Santa Cruz Biotechnology (CA, USA)	Sc-5279
E-Cadherin	Mouse	1:200	-	Becton Dickinson	
Cleaved caspase 3	Rabbit	1:200	-	Santa Cruz Biotechnology (CA, USA)	Sc-22171
SF1	Rabbit	1:1000	-	Kenichirou Morohashi (Kyushu, Japan)	
β-Catenin	Mouse monoclonal	1:100	14	Becton Dickinson	BD610153
Tyrosine hydroxylase	Rabbit	1:100	-	Cell Signaling (CA, USA)	2792
INSR	Rabbit	1:1000		Santa Cruz Biotechnology (CA, USA)	Sc-711
IGF1R	Rabbit	1:1000		Santa Cruz Biotechnology (CA, USA)	Sc-712
Actin	Mouse monoclonal	1:1000	C-4	Chemicon (Milipore)	MAB1501
KI-67	Mouse	1:100		Becton Dickinson	

Secondary antibodies:

Protein targeted	Host	Dilution	Provider	Catalogue number
HRP-conjugated anti-mouse antibodies	Goat		Biorad (Basel, Switzerland)	170-6516
HRP-conjugated anti-rabbit antibodies	Goat		Biorad (Basel, Switzerland)	170-6515
HRP-conjugated anti-goat antibodies	Rabbit		Sigma (St Louis, MO)	A5420
Anti-guinea pig Alexa 488	Goat	1:500	Invitrogen (OR, USA)	A11073
Anti-rabbit Alexa 594	Goat	1:500	Invitrogen (OR, USA)	A11012
Anti-guinea pig phosphatase alkaline antibodies	Goat and Donkey	1:500	Jackson Immuno Research (PA, USA)	706-055-148

Anti-mouse biotinylated antibodies	Donkey	1:500	Jackson Immuno Research (PA, USA)	715-065-150

Other reagents:

Name	Provider	Catalogue number
Complete EDTA-free inhibitors	Roche (Basel, Switzerland)	11873580001
ECL reagent	Amersham (Dübendorf, Switzerland)	RPN2135
Nitrocellulose membranes	Amersham (Dübendorf, Switzerland)	RPN303D
Trizol Reagent	Invitrogen (Carlsbad, CA)	15596-018
random hexamer primers	Invitrogen (Carlsbad, CA)	48190-011
SuperScript II Reverse Transcriptase	Invitrogen (Carlsbad, CA)	18064-022
SYBR green PCR master mix	Applied Biosystems	4309155
BCA protein assay kit	Pierce (Lausanne, Switzerland)	
RNeasy Micro Kit	Qiagen (Hombrechtikon, Switzerland)	74004
MessageAmpTM II-Biotin Enhanced Single Round aRNA Amplification Kit	Ambion	AM1791
Mouse Genome 430 2.0 Arrays	Affymetrix, High Wycombe, UK	
TUNEL reaction	Roche Applied Science	11767305001
