# Supplementary material for: Insulin and IGF1 Receptors Are Essential for XX and XY Gonadal Differentiation and Adrenal Development in Mice
Source: PLoS Genet. 2013 Jan 3;9(1):e1003160. doi: 10.1371/journal.pgen.1003160 (PMC3536656; doi:10.1371/journal.pgen.1003160)
Supplement: Table S4 — Primer sequences used for Real-Time PCR. (PDF) [file pgen.1003160.s016.pdf]

SUPPLEMENTARY TABLE 4: Primer Sequences for Real-Time PCR

Mouse	Forward	Reverse
**Sf-1**	5’ CCTCTTCAGCCTCGATGTGAA 3’	5’ GGCAGCGGTTGGCCTTTT 3’
**Sry**	5’ TACAGGCTGCAGTTGCCTCA 3’	5’ TGTCCCACTGCAGAAGGTTGT 3’
**Sox9**	5’ GTACCCGCATCTGCACAAC 3’	5’ CTCCACGAAGGGTCTCTTCTC 3’
**Fgf9**	5′ TGCCAGAGAAACAGCCG 3′	5′ TCTTCAATCCATCCGATGCA 3′
**Amh**	5′ TAGTCCTACATCTGGCTGAGTGATATG 3′	5′ CCAGGTGGAGGCTCTTGGA 3′
**Ptgds**	5’ TCGACCGCAGCAAGCAA 3’	5’ TGAGGGACAGGTGCAGGTACT 3’
**Cyp11a1**	5’ CTGGGATGTGATTTTCAATAAAGCT 3’	5’ CAGTAGAAGTTCTGGGTGTACTCATCAG 3’
**Gys**	5’ TTGTCGGACTTGCTAGATTGGAA 3’	5’ CGCGCAGACATGTAGTACCG 3’
**Rspo1**	5’ CCGCTAACAGCACCATGGA 3’	5’ CGCTCATTTCACATTGTGCAG 3’
**Wnt4**	5’ CGCGAGCAATTGGCTGTAC 3’	5’ GAGATGCTGCCCACCGAT 3’
**Foxl2**	5’ GAGCTCGCCATGATGCATT 3'	5’ GCCCGTCTTGCTGTCGTG 3’
**Lef1**	5’ CTACCACGACAAGGCCAGAGA 3’	5’ CCGTCTGGATGCTTTCCTTC 3’
**Insr**	5’ GGACCATGCCTGAAGCTAAG 3’	5’ GGACCATGCCTGAAGCTAAG 3’
**Igf1r**	5’ AGGCTGAGAAGCTGGGCTGCA 3’	5’ ACAGAAGCATACAGCACTCCA 3’
**Cyclophilin B**	5’ ATGTGGTTTTCGGCAAAGTT 3’	5’ TGACATCCTTCAGTGGCTTG 3’
**Tubulin beta**	5’ GCAGTGCGGCAACCAGAT 3’	5’ AGTGGGATCAATGCCATGCT 3’
**Actin beta**	5’ CTAAGGCCAACCGTGAAAAGAT 3’	5’ CACAGCCTGGATGGCTACGT 3’
**Tubulin a2**	5’ AGGAGCTGGCAAGCATGTG 3’	5’ CGGTGCGAACTTCATCGAT 3’
